# Adding non-randomised studies to a Cochrane review brings complementary information for healthcare stakeholders: an augmented systematic review and meta-analysis

**DOI:** 10.1186/s12913-016-1816-5

**Published:** 2016-10-21

**Authors:** Chantal Arditi, Bernard Burnand, Isabelle Peytremann-Bridevaux

**Affiliations:** 1 Institut Universitaire de Médecine Sociale et Préventive (IUMSP), Lausanne University Hospital, Rte de la Corniche 10, 1010 Lausanne, Switzerland; 2 Cochrane Switzerland, IUMSP, Lausanne University Hospital, Rte de la Corniche 10, 1010 Lausanne, Switzerland

**Keywords:** Systematic review, Meta-analysis, Asthma, Chronic disease management, Complex interventions, Study design

## Abstract

**Background:**

To reduce the burden of asthma, chronic disease management (CDM) programmes have been widely implemented and evaluated. Reviews including randomised controlled trials (RCTs) suggest that CDM programmes for asthma are effective. Other study designs are however often used for pragmatic reasons, but excluded from these reviews because of their design. We aimed to examine what complementary information could be retrieved from the addition of non-randomised studies to the studies included in a published Cochrane review on asthma CDM programmes, for healthcare stakeholders involved in the development, implementation, conduct or long-term sustainability of such programmes.

**Methods:**

Extending a previously published Cochrane review, we performed a systematic review (augmented review) including any type of study designs instead of only those initially accepted by Cochrane and the Effective Practice and Organization of Care Review group. After double data selection and extraction, we compared study and intervention characteristics, assessed methodological quality and ran meta-analyses, by study design.

**Results:**

We added 37 studies to the 20 studies included in the Cochrane review. The applicability of results was increased because of the larger variety of settings and asthma population considered. Also, adding non-randomised studies provided new evidence of improvements associated with CDM intervention (i.e. healthcare utilisation, days off work, use of action plan). Finally, evidence of CDM effectiveness in the added studies was consistent with the Cochrane review in terms of direction of effects.

**Conclusions:**

The evidence of this augmented review is applicable to a broader set of patients and settings than those in the original Cochrane review. It also strengthens the message that CDM programmes have a beneficial effect on quality of life and disease severity, meaningful outcomes for the everyday life of patients with asthma. Despite the moderate to low methodological quality of all studies included, calling for caution in results interpretation and improvements in CDM evaluation methods and reporting, the inclusion of a broader set of study designs in systematic reviews of complex interventions, such as chronic disease management, is likely to be of high value and interest to patients, policymakers and other healthcare stakeholders.

**Electronic supplementary material:**

The online version of this article (doi:10.1186/s12913-016-1816-5) contains supplementary material, which is available to authorized users.

## Background

Asthma is a global health problem affecting over 300 million people worldwide [1] and a major cause of disability, poor quality of life, health resources utilisation, and costs [2–4]. Despite the availability of effective therapies, asthma control remains suboptimal, calling for the implementation and evaluation of effective asthma management interventions [5]. These include chronic disease management (CDM) programmes, which are more than simple patient education, encompassing a set of coherent interventions that centres on the patients’ needs, focuses on education and self-management, on healthcare integration and coordination provided by various professionals, as well as on improvements in communication between patients and healthcare providers [6–12].

Summarising the evidence of CDM programmes for asthma in a systematic review is not an easy task [13]. Most often, state-of-the art Cochrane systematic reviews of interventions include randomised controlled trials (RCTs) only [14]. However, RCTs are often not the most suitable design for evaluating complex and context-dependent interventions [15–17]. Non-randomised studies, such as controlled and uncontrolled before-after studies, are often undertaken in the pragmatic context of CDM programme implementation in communities [16]. These non-randomised studies could be very informative for the various healthcare stakeholders involved in the development, implementation, conduct and long-term sustainability of such programmes, especially in terms of contexts, key components and implementation processes. Including non-randomised studies (NRS) or non-controlled studies (NCS) in systematic reviews is challenging, however, notably when assessing study quality and the potential for selection bias and its impact on study results [14, 18]. Yet, reviews and meta-reviews have shown that non-randomised studies do not systematically lead to biased results and that there is little evidence for significant effect estimate differences between observational studies and RCTs [14, 19].

The main objective of this systematic review was to examine what complementary information could be retrieved from the addition of NRS and NCS to a published Cochrane review on asthma CDM programmes, for healthcare stakeholders involved at various levels in the development, implementation, conduct and long-term sustainability of such programmes. We also assessed how the effectiveness of asthma CDM programmes varied across included study designs.

## Methods

This systematic review (augmented review throughout the text) is an extension of a previously published Cochrane review [20] evaluating the effectiveness of CDM programmes in adults with asthma, that was conducted independently from Cochrane. We used the same methodology as in the Cochrane review, except for study designs considered, risk of bias assessments and some aspects of the statistical analyses. While major methodological elements are presented thereafter, details of the methodology of the published Cochrane review are available in the original text [20].

### Identification of studies and eligible study design

In the original review, we searched the following databases: MEDLINE, EMBASE, CINHAL, PSYCINFO, CENTRAL Cochrane and the Specialized Registers of the Effective Practice and Organisation of Care (EPOC) and Airways Disease Groups up to June 2014 [20]. We also hand-searched reference lists of retrieved papers and relevant narrative or systematic reviews, and registers of clinical trials to identify new and ongoing trials. Language restrictions were not applied. Eligible study designs in the original review were RCTs, quasi-randomised controlled trials (QRCTs) (i.e. an experimental study in which participants are prospectively allocated using a method that is not random), controlled before-after studies (CBA*s) (i.e. a study in which observations are made before and after the implementation of an intervention, both in a group that receives an intervention and in a control group that does not, with at least two intervention and two control sites) and interrupted time series (ITS*s) (i.e. a study that uses observations at multiple time points before and after an intervention, with at least three data points available before and after the intervention), complying with the guidelines from the EPOC Review group [21]. In this augmented review, we did not run a new search but modified the design inclusion criteria to also include any CBAs, any ITSs, before-after studies without an external control group (BAs) (i.e. outcomes are measured before and after the implementation of an intervention, in a single group) and cross-sectional studies (XSs) (i.e. concurrent measurement of intervention and outcomes), identified in the original search of the Cochrane review and excluded because of the study design (*n* = 66). We excluded studies presenting secondary analyses and implementation results only.

### Types of interventions and outcomes

We included CDM programmes targeting adult participants with a diagnosis of asthma in both reviews, if the following five criteria were all met: 1) at least one organisational component targeting patients; 2) at least one organisational component targeting healthcare professionals and/or the healthcare system; 3) presence of patient education and/or self-management support component; 4) active involvement of two or more healthcare professionals in the patient’s care; 5) minimum duration of 3 months of at least one of the criteria one to three.

In both reviews we looked at the same organisational and patient-level outcomes; the ten primary outcomes, pre-defined in the Cochrane protocol [20], were also considered in this augmented review. Organisational outcomes included organisation of care outcomes, process outcomes (use of an action plan), and healthcare use outcomes (hospitalisations, unscheduled visits to an emergency department or physician office). Patient-level outcomes included quality of life (QoL) outcomes (asthma-specific QoL), symptoms and activity level outcomes (asthma severity, asthma exacerbations, days off work/school), self-care outcomes (self-efficacy scores), lung function outcomes (forced expiratory volume in one second (FEV1) or peak expiratory flow rate (PEFR)), and patient satisfaction.

### Data extraction

Two reviewers, working independently and in duplicate, re-assessed the previously excluded articles (*n* = 66) for eligibility criteria and extracted data regarding study design, country and healthcare setting, CDM intervention (number of and main component, comprehensiveness of intervention -eight or more components-, duration of the longest component), patient characteristics (age, sex, asthma severity) and reported outcomes (structure indicators, process of care and intermediate measures, clinical effect). Where required, we sought additional information by contacting authors.

### Assessment of risk of bias

We assessed the methodological quality of the studies using the Cochrane Collaboration’s tool modified by EPOC [22] for assessing risk of bias for RCTs, QRCTs, and CBAs, used in the original review, with additional items for BAs, ITSs and XSs extracted from various instruments: the new Cochrane risk of bias assessment tool for non-randomised studies of interventions (ACROBAT-NRSI) [23], the checklist for the assessment of the methodological quality of both randomised and non-randomised studies of health care interventions by Downs and Black [24], and the quality assessment tool for quantitative studies by the Effective Public Health Practice Project (EPHPP) [25, 26]. Domains of bias included in the final instrument, selected by consensus among authors to discriminate the quality of all study designs and encompass all relevant bias domains, were 1) random sequence generation, 2) allocation concealment, 3) baseline characteristics similar, 4) baseline outcomes similar, 5) confounding unlikely, 6) appropriate analyses (e.g. adjusted or time trend analyses if required), 7) sample representative of source population, 8) intervention independent of other changes, 9) intervention integrity (e.g. intervention delivered as intended and consistently, adequate protection against contamination), 10) blinding of outcome assessment, 11) incomplete outcome data addressed, and 12) free of other bias (see Additional file 1 for the risk of bias extraction form). Any disagreement was resolved by discussion or involvement of an arbitrator, or both.

### Measures of treatment effect and data synthesis

Measures of treatment effect were reported separately by study design, as suggested by recent guidance on including NRS in systematic reviews [27]. For RCTs, we reported results of dichotomous outcomes as odd ratios (OR) and results of continuous outcomes as mean differences (MD) or standardized mean differences (SMD) if different instruments or scales were used for a scale-type outcome, using post intervention (follow-up) values. SMDs were calculated by dividing the difference in mean scores between the intervention and control group by an estimate of the (pooled) standard deviation, resulting in a ‘scale free’ estimate of the effect for each study which can be pooled across studies regardless of the scale of measurement used in each study. Using rules of thumb, SMDs lower than 0.4 represent a small effect, SMDs between 0.4 and 0.7 represent a moderate effect, and SMDs higher than 0.7 represent a large effect [28]. For QRCTs, CBA*s, and CBAs, we intended to report results derived from statistical analyses adjusting for baseline measures. However, as adjusted results were not available in the included studies, we reported OR, MD or SMD using follow-up values only. CBA*s and CBAs were analysed in the same study design group. For XSs, we also reported unadjusted OR, MD and SMD as adjusted results were not available. For BAs, we reported OR and MD or SMD, considering the baseline data as data for the “control” group. For ITSs, we planned to compare time trends before and after the intervention, or to re-analyse the data if analyses in the original paper were inappropriate, according to EPOC guidance [29]. However, because of the lack of reported data in the ITSs, re-analysis was not possible and we considered ITS data as BA data.

We reported the number of times the ten primary outcomes were assessed, and the number of times that statistically significant results favouring CDM were found by authors (for at least one dimension in case of scale outcomes), by study design. Then, where possible, we conducted meta-analyses using the Cochrane Review Manager software [30] to calculate the overall effect size for all relevant outcomes. We pooled results of studies of similar design using the random-effects model [21] to incorporate some level of expected heterogeneity among pooled studies. All results were expressed with 95 % confidence intervals, and statistical heterogeneity among trials was examined with Cochran’s Q test and by calculating the I^2^ statistic describing the proportion of variability in the summary estimate that is due to heterogeneity rather than by chance.

## Results

**Fig. 1:**
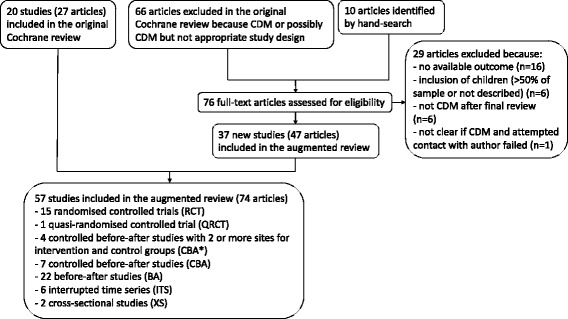
Flow diagram. CDM: chronic disease management

Of the 10,593 records identified in the Cochrane review, 20 studies (from 27 articles) met all inclusion criteria and 66 articles were excluded because of the study design. Of these 66 excluded articles, 29 were rejected because no outcomes were available, children represented more than 50 % of the sample, or the intervention did not fulfil the inclusion criteria after further examination, leading to 37 remaining articles to additionally consider in the augmented review. Ten other articles were found by hand searching the reference lists of included articles. We therefore included 27 Cochrane articles and 47 additional articles, for a total of 57 studies, from 74 articles, in this augmented review (see flow diagram in Fig. 1 and key components of all included studies in the supplementary table (see Additional file 2)). These 57 studies consisted of seven CBAs [31–37], 22 BAs [38–59], six ITSs [60–65] and two XSs [66, 67], in addition to the 15 RCTs [68–82], one QRCT [83, 84], and four CBA*s [85–88] of the Cochrane review.

### Characteristics of included studies

#### Programme and recruitment setting

Details of the 57 studies are presented in Table 1 and in the supplementary table (see Additional file 2: Supplementary Table). While about half of the RCTs and CBA*s in the Cochrane review were carried out in North America (USA and Canada), only 30 % of the studies added in this augmented review were held in that region. These studies were carried out more often in Europe (Finland, Germany, Netherlands, Russia, Spain, Sweden, Switzerland, UK) and other parts of the world (Australia, Brazil, India, New Zealand, Thailand) compared to the countries represented in the RCTs, QRCT and CBA*s (Denmark, France, Germany, Netherlands, UK and Australia, Japan, Taiwan). Also, they recruited patients from a larger variety of patient pools: five studies recruited patients from the general population compared to none in the Cochrane studies and a higher percentage of studies recruited patients in specialised clinics or outpatient hospital departments. Finally, care was delivered proportionally more often in specialised clinics or outpatient hospital departments in the studies added in this augmented review, compared to studies in the Cochrane review, which were mostly community-based.

**Table 1:** Study and intervention characteristics, by study design

	*Studies in Cochrane review*			*Newly added studies*			
	RCT	QRCT	CBA*	CBA	BA	ITS	XS
	*N* = 15	*N* = 1	*N* = 4	*N* = 7	*N* = 22	*N* = 6	*N* = 2
Study characteristics:							
Publication year, mean (SD)	2004.0 (5.3)	2001	2004.8 (4.1)	2003.9 (6.1)	2002.8 (5.7)	2009.8 (4.0)	2006.5 (6.4)
Region, *n* (%)							
Europe	4 (26.7 %)	Denmark	1 (25.0 %)	0	9 (40.9 %)	3 (50.0 %)	Sweden
North America	8 (53.3 %)	-	2 (50.0 %)	4 (57.1 %)	6 (27.3 %)	1 (16.7 %)	0
Other	3 (20.0 %)	-	1 (25.0 %)	3 (42.9 %)	7 (31.8 %)	2 (33.3 %)	Brazil
Location of care, *n* (%)							
Outpatient care^a^	3 (20.0 %)	-	0	2 (28.6 %)	9 (40.9 %)	3 (50.0 %)	0
Community-based care^b^	6 (40.0 %)	1	3 (75.0 %)	3 (42.9 %)	10 (45.5 %)	1(16.7 %)	2 (100 %)
Mixed	6 (40.0 %)	-	1 (25.0 %)	2 (28.6 %)	3 (13.6 %)	2 (33.3 %)	0
Recruitment pool, *n* (%)							
General population	0	-	0	0	2 (9.1 %)	3 (50.0 %)	1 (50 %)
Insurance clients	2 (13.3 %)	-	3 (75.0 %)	2 (28.6 %)	1 (4.5 %)	1 (16.7 %)	0
PCP/Pharmacy	5 (33.3 %)	1	1 (25.0 %)	2 (28.6 %)	7 (31.8 %)	0	1 (50 %)
Outpatients^c^	3 (20.0 %)	-	0	2 (28.6 %)	8 (36.3 %)	2 (33.3)	0
Inpatients	3 (20.0 %)	-	0	0	0	0	0
Multisector	2 (13.3 %)	-	0	1 (14.3 %)	4 (18.2 %)	0	0
Patients, mean (SD)	157.7 (153.1)	413	19742 (34137.3)	683.0 (1157.5)	347.4 (725.2)	32553 (16363.9)	330.5 (24.7)
Median	96	-	3717	239.5	100.0	32553	330.5
Age, mean (SD)	43.0 (6.3)	40.6	34.0 (3.6)	42.3 (5.4)	42.2 (6.8)	nr	48.5
Women, mean % (SD)	61.3 (14.7)	56.2 %	52.8 (10.6)	71.8 (4.5)	66.6 (11.1)	nr	55.5 (2.3)
Asthma severity, n (%)							
Mild-moderate	1 (6.7 %)	-	0	0	7 (31.8 %)	0	1 (50 %)
Moderate-severe	11 (73.3 %)	1	0	4 (57.1 %)	10 (45.5 %)	0	0
Not reported	3 (20.0 %)	-	4 (100.0 %)	3 (42.9 %)	5 (22.7 %)	6 (100 %)	1 (50 %)
FEV1, mean (SD)	64.6 (20.9)	nr	nr	nr	79.4 (10.4)	nr	nr
ICS use, mean % (SD)	73.8 (32.0)	nr	nr	74.4 (26.5)	52.0 (30.3)	52.8 (36.4)	44.7 %
Intervention characteristics:							
Number of intervention components, mean (SD)	8.5 (2.5)	9	7.8 (2.5)	7.1 (1.2)	6.7 (2.0)	10.0 (2.1)	7
Comprehensive intervention (≥8 components), *n* (%)	8 (53.3 %)	1	2 (50.0 %)	3 (42.9 %)	5 (22.7 %)	5 (71.4 %)	0
Main component, *n* (%)							
Educational	7 (46.7 %)	-	1 (25.0 %)	6 (85.7 %)	14(63.6 %)	0	1 (50 %)
Org_healthcare	2 (13.3 %)	-	2 (50.0 %)	1 (14.3 %	6 (27.3 %)	5 (71.4 %)	1 (50 %)
Org_patient	1 (6.7 %)	1	0	0	0	0	0
Multicomponent	5 (33.3 %)	-	1 (25.0 %)	0	2 (9.1 %)	1 (16.7 %)	0
Duration of intervention (months), mean (SD)	7.5 (3.2)	12	22.5 (21.0)	12.5 (12.6)	13.1 (8.0)	ongoing	4

*RCT* randomised controlled trial, *QRCT* quasi-randomised controlled trial, CBA* controlled before-after study (with at least two intervention and two control sites), *CBA* controlled before-after study, *ITS* interrupted-times-series study, *BA* before-after study (without external control group), *XS* cross-sectional study (with concurrent measurement of intervention exposure and outcomes), *PCP* primary care practice, *SD* standard deviation, *FEV1* forced expiratory volume in 1 s (% predicted), *ICS* inhaled corticosteroids, *Org* organisational, *nr* not reported

^a^Outpatient care: ambulatory care provided by specialised clinic or hospital

^b^Community-based care: care provided at a primary care practice, pharmacy or health unit

^c^Outpatients: patients seen by specialised clinic or outpatient hospital department

#### Study population

A total of 158,907 patients (between 25 and 70,900 per study, median: 111) were included in the 51 studies reporting this information, with a mean number of patients included in the studies that differed according to study design (see Table 1): RCTs included less patients on average compared to the other designs, while all ITSs included over 10,000 patients. Patients’ mean age ranged from 34.0 in the CBA*s to 48.5 in the XS reporting this information, and the mean percentage of women included in the studies ranged from 53 % in the CBA*s to 72 % in the CBAs. Compared with patients in RCTs, those included in BAs appeared to suffer from less severe asthma: the percentage of studies including patients suffering from mild to moderate asthma was higher in studies reporting this information, the mean FEV1 was higher, and the mean percentage of patients using inhaled corticosteroids (ICS) was lower.

#### Interventions

The mean number of independent components per CDM intervention was highest in the ITSs, which were also more often defined as comprehensive interventions (including at least eight components), while BAs included fewer components and were less often comprehensive (see Table 1). Education and/or self-management support was the main component in most CBAs and BAs, while the RCTs were more often multi-component. The three components most often implemented in RCTs were individual education sessions (100 % of RCTs), explicit teamwork and collaborative processes between healthcare providers (87 % of RCTs) and structured follow-up (80 % of RCTs), while the three most frequent components in the QRCT and CBA*s were individual education sessions (80 % of studies), structured follow-up (80 % of studies), reminders, feedback and other routine reporting sheets (80 % of studies). In studies added in this augmented review, the three most frequent components were structured follow-up (68 % of studies), healthcare professionals education (68 % of studies) and providing an action plan (62 % of studies). The RCTs evaluated shorter interventions on average (mean duration less than 12 months) compared to the other designs. Thirteen of the newly added studies were ongoing (without time limitation), while none of the studies in the Cochrane review was ongoing.

#### Outcome measures

A large variety of outcomes were reported in the studies (all reported outcomes are listed in the supplementary table (see Additional file 2: Supplementary Table)). While RCTs reported more often self-reported scale outcomes (i.e. score on asthma-specific QoL, asthma severity, asthma knowledge and self-care), the studies added in this augmented review reported more often healthcare use and days off work/school. In addition, while the four outcomes most often measured in RCTs were asthma-specific quality of life (80 % of RCTs), medication use and ICS prescription (60 % of RCTs), hospitalisations (53 % of RCTs) and asthma severity (53 % of RCTs), the four outcomes most often reported in the QRCT and CBA*s were hospitalisations (all five studies), unscheduled visits (60 % of studies), general practitioner visits (60 % of studies) and ICS prescription (60 % of studies). In the studies added in this augmented review, the four outcomes most often reported were hospitalisations (in 65 % of studies), unscheduled visits (62 % of studies), ICS prescription (38 % of studies) and delivery of intervention (38 % of studies).

#### Risk of bias

**Fig. 2:**
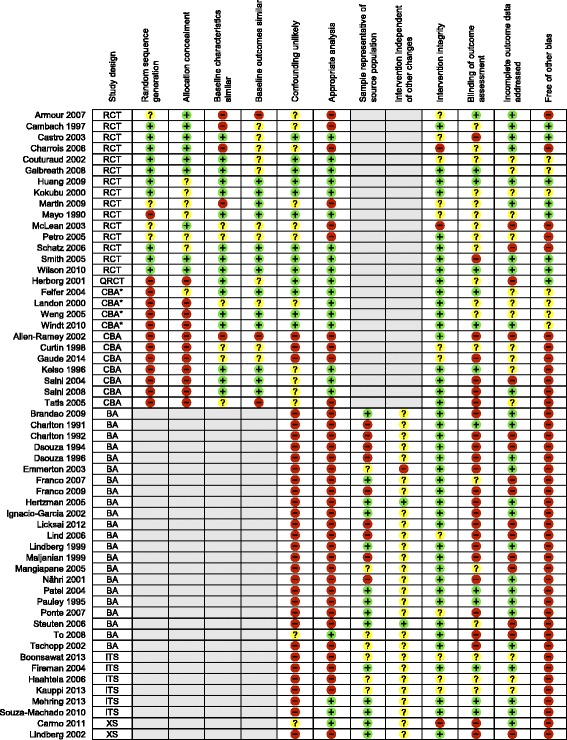
Risk of bias by study design.  Low risk of bias;  Unclear risk of bias;  High risk of bias;  Not applicable; RCT: randomised controlled trial; QRCT: quasi-randomised controlled trial; CBA*: controlled before-after study (with at least two intervention and two control sites); CBA: controlled before-after study; ITS: interrupted-times-series study; BA: before-after study (without external control group); XS: cross-sectional study (with concurrent measurement of intervention exposure and outcomes)

In the Cochrane review, the quality of the evidence was rated as moderate to low based on the GRADE approach [89] , despite the fact that most studies were RCTs. This was mainly due to study design limitations, which resulted in either unclear or high risk of bias in most cases and in wide confidence intervals. Detail assessment can be found in the Cochrane publication [20] and in Fig. 2.

In this augmented review, the overall methodological quality of the newly added studies was rated as low (see Fig. 2 for risk of bias assessment per study). More specifically, only two of the seven CBAs attempted to minimize confounding bias by stratifying patients according to asthma severity [31] and matching the control group for age, gender, onset of asthma, healthcare use, and employment [34]. Also, across all designs, most studies were at high risk of confounding bias and did not run appropriate analyses. For instance, only two ITSs performed appropriate trend analyses, using the Cochran Armitage test for trend [64] and linear regression modelling [65]. In addition, patients were deemed representative of the source population (i.e. included patients comprising the entire source population, an unselected sample of consecutive patients or a random sample) in less than half of the included studies and most studies were at unclear risk of bias regarding the independence of the intervention from other changes. Lastly, while more than half of the studies were at high risk of detection bias (outcomes not assessed blindly), about half of the studies were at low risk of attrition bias (less than 20 % of patients lost to follow-up) and most studies were at low risk of intervention integrity (interventions delivered as intended and consistently).

### Effects of asthma CDM programmes

We report in the following section the effectiveness results for the pre-defined ten primary outcomes.

Among the 57 included studies of this augmented review, 17 studies did not contribute to any meta-analysis, because of data format heterogeneity (e.g. continuous versus dichotomous data, change from baseline versus follow-up data only) and data unavailability.

#### Overview

Table 2 displays the number of studies reporting statistically significant effects associated with CDM for the nine primary outcomes (no data was found for asthma exacerbations, one of the primary outcomes), among studies reporting the outcome of interest, by study design. Across all outcomes, the RCTs, QRCT and CBA*s of the Cochrane review had lower proportion of outcomes that significantly improved in response to CDM (49.1, 33.3 and 44.4 %, respectively) compared to CBAs, BAs, ITSs and XSs (80.0, 72.4, 50.0 and 80.0 %, respectively). Across all designs, outcomes that significantly improved more often were asthma self-efficacy (91.7 %), asthma-specific QoL (81.8 %), asthma severity (80.0 %) and use of an action plan (80.0 %), while outcomes that improved less often were patient satisfaction (28.6 %), lung function (40.7 %), days off work or school (53.3 %) and healthcare use (hospitalisation 51.6 %, unscheduled visit 59.4 %). We present below the meta-analysis results (if available) for the ten primary outcomes, by order of a priori defined clinical significance.

**Table 2:** Study effects, by study design

Number of studies reporting SS effect / number of studies reporting outcome	*Studies in Cochrane review*			*Newly added studies*			
	RCT	QRCT	CBA*	CBA	BA	ITS	XS
	*N* = 15	*N* = 1	*N* = 4	*N* = 7	*N* = 22	*N* = 6	*N* = 2
Asthma-specific quality of life	9 / 12	1 / 1	1 / 1	2 / 3	5 / 5	-	-
Hospitalisations	3 / 6	0 / 1	0 / 2	3 / 4	8 / 13	3 / 6	0 / 1
ED or unscheduled visits	1 / 6	0 / 1	0 / 2	3 / 4	13 / 16	1 / 2	1 / 1
Asthma self-efficacy	4 / 5	-	1 / 1	2 / 2	3 / 3	-	1 / 1
Asthma severity	5 / 8	1 / 1	-	2 / 2	6 / 7	1 / 1	1 / 1
Days off school or work	0 / 2	-	0 / 1	0 / 1	8 / 11	-	-
Use of an action plan	1 / 2	-	2 / 2	3 / 3	4 / 5	1 / 2	1 / 1
Patient satisfaction	0 / 2	0 / 1	-	-	2 / 3	0 / 1	-
Lung function	4 / 12	0 / 1	-	1 / 1	6 / 13	-	-

*RCT* randomised controlled trial, *QRCT* quasi-randomised controlled trial, CBA* controlled before-after study (with at least two intervention and two control sites), *CBA* controlled before-after study, *ITS* interrupted-times-series study, *BA* before-after study (without external control group), *XS* cross-sectional study (with concurrent measurement of intervention exposure and outcomes), *SS* statistically significant, *ED* emergency department

#### Asthma-specific quality of life

**Fig. 3:**
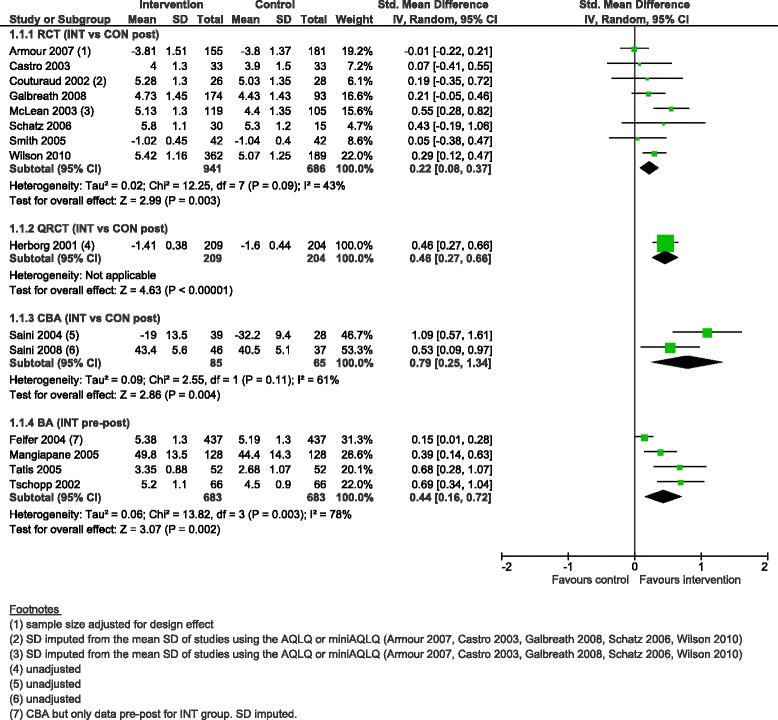
Asthma-specific quality of life score, forest plot by study design. RCT: randomised controlled trial; QRCT: quasi-randomised controlled trial; CBA: controlled before-after study; BA: before-after study (without external control group); INT: intervention; CON: control; SD: standard deviation; IV: inverse variance; CI: confidence interval

CDM programmes resulted in improved asthma-specific QoL scores in all designs, with a small effect in RCTs (SMD 0.22, 95 % CI 0.08 to 0.37, 8 studies), moderate effect in BAs (SMD 0.44, 95 % CI 0.16 to 0.72, 4 studies) and the QRCT (SMD 0.46, 95 % CI 0.27 to 0.66, 1 study), and large effect in CBAs (SMD 0.79, 95 % CI 0.25 to 1.34, 2 studies) (Fig. 3).

#### Healthcare use: hospitalisations and unscheduled visits

**Fig. 4:**
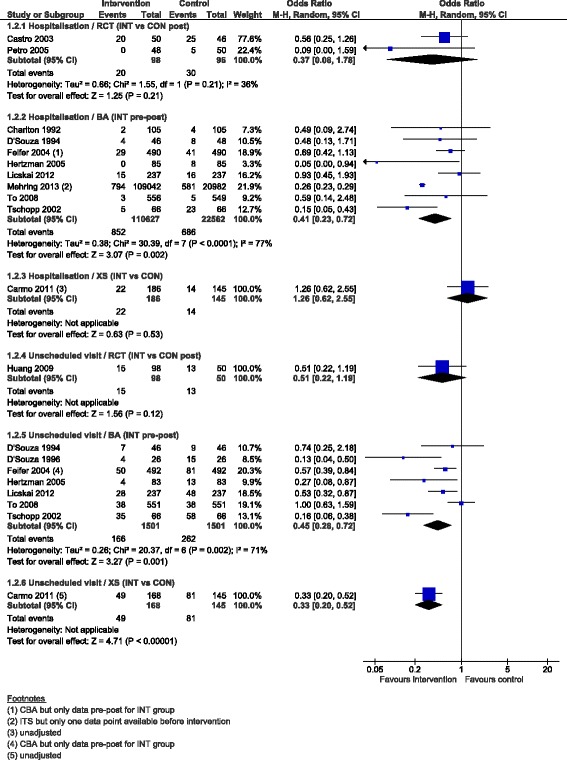
Percentage patients with ≥1 hospitalisation and ≥1 unscheduled visit, forest plot by study design. RCT: randomised controlled trial; BA: before-after study (without external control group); XS: cross-sectional study (with concurrent measurement of intervention exposure and outcomes); INT: intervention; CON: control; M-H: Mantel-Haenszel; CI: confidence interval

While we were not able to perform meta-analysis on healthcare use data in the Cochrane review because of the wide variability in means and rates at baseline, length of follow-up, data type and reasons for healthcare use (asthma-specific or all-cause), we were able in this augmented review to run meta-analyses on the percentage of patients with one or more hospitalisation and one or more unscheduled visits (Fig. 4). Only the meta-analyses for the BAs showed a significant lower likelihood of hospitalisation and unscheduled visit at follow-up compared to baseline (OR 0.41, 95 % CI 0.23 to 0.72, 8 studies and OR 0.45, 95 % CI 0.28 to 0.72, 7 studies, respectively).

#### Asthma exacerbations

Meta-analysis on asthma exacerbations, defined as prompting hospitalisation, an unscheduled or ED visit, or systemic rescue glucocorticoids, was not possible as data were not reported as such in the included studies.

#### Self-efficacy

**Fig. 5:**
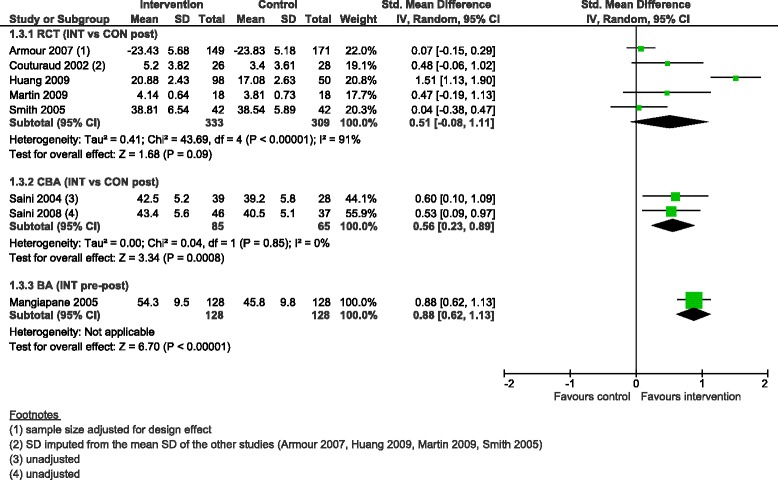
Self-efficacy score, forest plot by study design. RCT: randomised controlled trial; CBA: controlled before-after study; BA: before-after study (without external control group); INT: intervention; CON: control; SD: standard deviation; IV: inverse variance; CI: confidence interval

CDM programmes were associated with improvement in asthma self-efficacy scores, with a moderate, inconclusive and heterogeneous effect in RCTs (SMD 0.51, 95 % CI −0.08 to 1.11, 5 studies, I^2^ = 91 %), a moderate effect in CBAs (SMD 0.56, 95 % CI 0.23 to 0.89, 2 studies) and a large effect in the BA (SMD 0.88, 95 % CI 0.62 to 1.13, 1 study) (Fig. 5).

#### Asthma severity

**Fig. 6:**
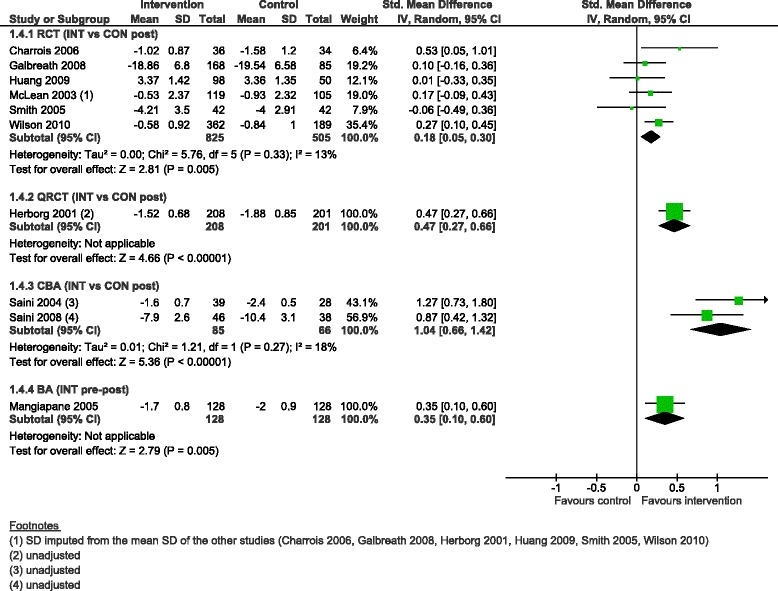
Asthma severity score, forest plot by study design. RCT: randomised controlled trial; QRCT: quasi randomised controlled trial; CBA: controlled before-after study; BA: before-after study (without external control group); INT: intervention; CON: control; SD: standard deviation; IV: inverse variance; CI: confidence interval

Asthma severity scores improved in all designs, with a small effect in RCTs (SMD 0.18, 95 % CI 0.05 to 0.30, 6 studies) and in the BA (SMD 0.35, 95 % CI 0.10 to 0.60, 1 study), a moderate effect in the QRCT (SMD 0.47, 95 % CI 0.27 to 0.66, 1 study), and large effect in the CBAs (SMD 1.04, 95 % CI 0.66 to 1.42, 2 studies) (Fig. 6).

#### Days off work or school

**Fig. 7:**
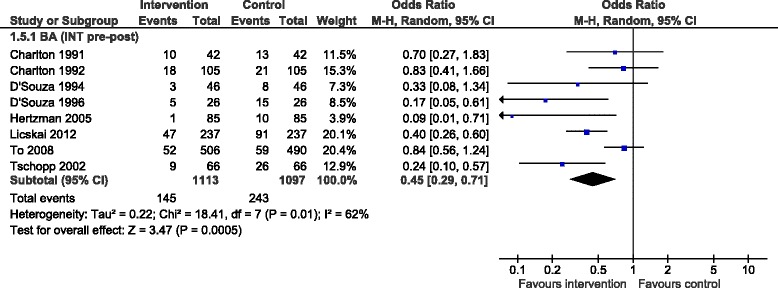
Percentage patients who missed ≥1 day off work or school, forest plot by study design. BA: before-after study (without external control group); INT: intervention; CON: control; M-H: Mantel-Haenszel; CI: confidence interval

While we were not able to pool data on days off work or school in the Cochrane review because of their heterogeneous format, we were able to pool BAs reporting the percentage of patients who missed at least one day of work or school due to asthma; it showed a moderate reduction after the intervention (OR 0.45, 95 % CI 0.29 to 0.71, 8 studies) (Fig. 7).

#### Use of an action plan

**Fig. 8:**
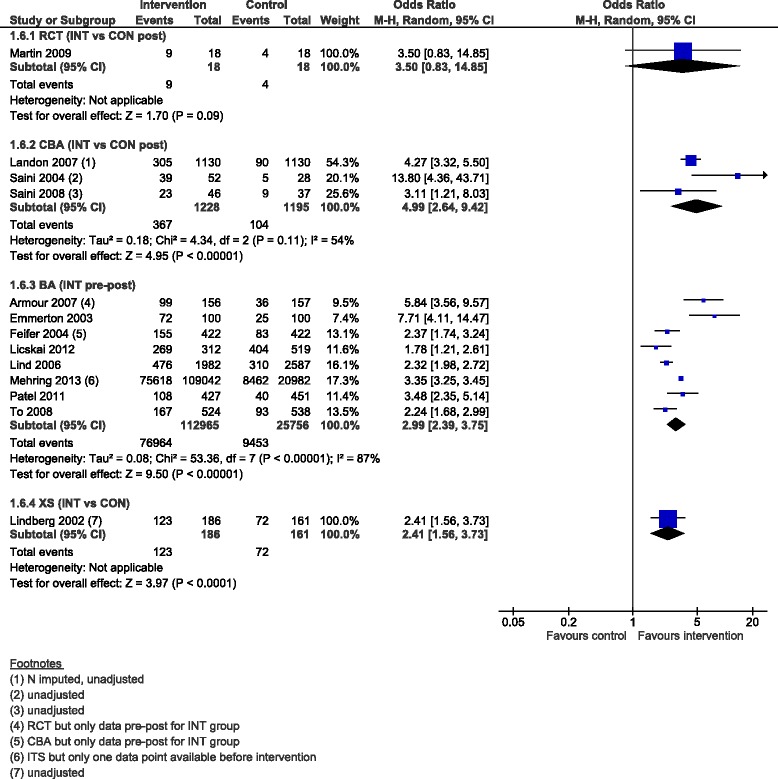
Percentage patients with an action plan, forest plot by study design. RCT: randomised controlled trial; CBA: controlled before-after study; BA: before-after study (without external control group); XS: cross-sectional study (with concurrent measurement of intervention exposure and outcomes); INT: intervention; CON: control; M-H: Mantel-Haenszel; CI: confidence interval

The pooled OR for the percentage of patients with an action plan at follow-up ranged from 2.41 (95 % CI 1.56 to 3.73) for the XS, 2.99 (95 % CI 2.39 to 3.75) for the eight BAs with a high degree of heterogeneity (I^2^ = 87 %), 3.50 (95 % CI 0.83 to 14.85) for the RCT, to 4.99 (2.64 to 9.42) for the three CBAs (Fig. 8).

#### Patient satisfaction

Due to the heterogeneity of patient satisfaction measures, we were unable to perform meta-analysis on patient satisfaction.

#### Lung function

**Fig. 9:**
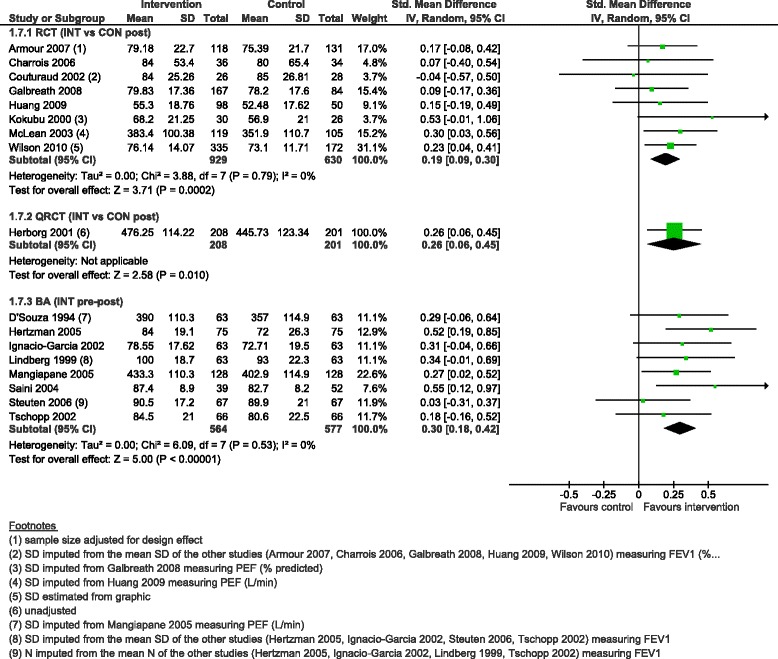
Lung function (FEV1 or PEF, in % predicted value or L/min), forest plot by study design. RCT: randomised controlled trial; QRCT: quasi randomised controlled trial; BA: before-after study (without external control group); INT: intervention; CON: control; SD: standard deviation; IV: inverse variance; CI: confidence interval

CDM programmes were associated with improvement in lung function (FEV1 or PEF value reported as per cent of predicted value or L/min) in all designs, with a small effect in all designs (SMD 0.19, 95 % CI 0.09 to 0.30 in eight RCTs, SMD 0.26, 95 % CI 0.06 to 0.45 in the QRCT, SMD 0.30, 95 % CI 0.18 to 0.42 in eight BAs) (Fig. 9).

#### Secondary outcomes

By adding NRS and NCS, we were able to examine one secondary outcome and run two additional meta-analyses on two secondary outcomes reported in sufficient studies to allow comment and analysis in the following paragraphs.

The impact of CDM programmes on the frequency of GP visits was bi-directional. One RCT found less frequent GP visits in the intervention group compared to the control group [78], while one QRCT [83] and one CBA [87] found more frequent GP visits. Two BAs found more frequent GP visits per patient at follow-up compared to baseline [44, 45], while five BAs and one ITS found on the contrary that the number of GP visits par patient decreased in the intervention group compared to baseline [39–42, 47, 61].

**Fig. 10:**
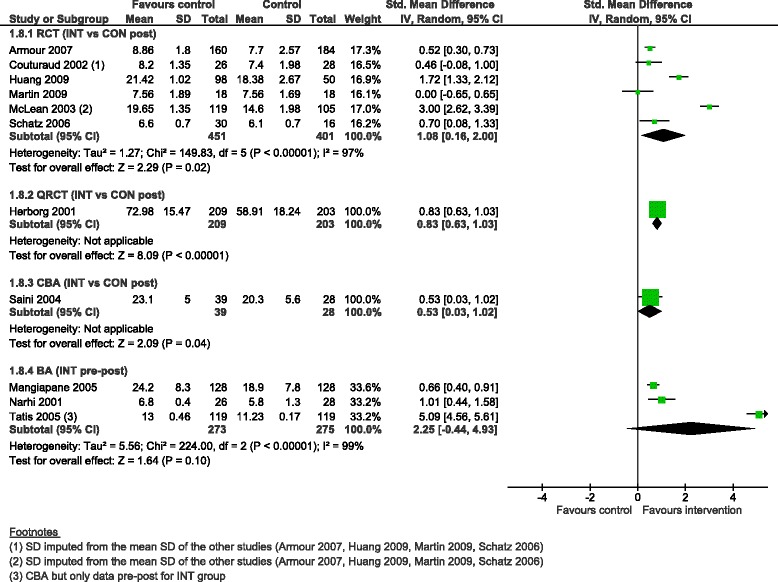
Asthma knowledge score, forest plot by study design. RCT: randomised controlled trial; QRCT: quasi randomised controlled trial; CBA: controlled before-after study; BA: before-after study (without external control group); INT: intervention; CON: control; SD: standard deviation; IV: inverse variance; CI: confidence interval

The pooled SMD for asthma knowledge ranged from 0.53 (95 % CI 0.03 to 1.02) for the CBA, 0.83 (95 % CI 0.63 to 1.03) for the QRCT, 1.08 (95 % CI 0.16 to 2.0) for six RCTs with a high degree of heterogeneity (I^2^ = 97 %), to 2.25 (95 % CI −0.44 to 4.93) for three BAs with a high degree of heterogeneity as well (I^2^ = 99 %) (Fig. 10).

**Fig. 11:**
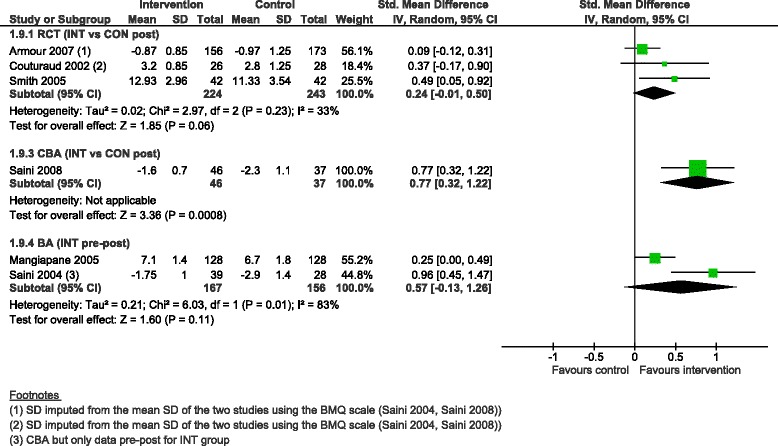
Compliance with medication score, forest plot by study design. RCT: randomised controlled trial; CBA: controlled before-after study; BA: before-after study (without external control group); INT: intervention; CON: control; SD: standard deviation; IV: inverse variance; CI: confidence interval

The pooled SMD for medication compliance scores ranged from 0.24 (95 % CI −0.01 to 0.50) for three RCTs, 0.57 (95 % CI −0.13 to 1.26) for two BAs with a high degree of heterogeneity (I^2^ = 83 %), to 0.77 (95 % CI 0.32 to 1.22) for the CBA (Fig. 11).

## Discussion

Including study designs other than RCTs, QRCTs and CBA*s allowed the addition of 37 studies to the 20 studies included in the Cochrane review. In terms of study and intervention characteristics, the newly added studies allowed the consideration of programmes taking place in proportionally more countries outside of North America that included population-wide interventions with greater number of patients in a greater variety of settings and patient pools, and were of longer duration. In addition, patients in the studies of this augmented review suffered from less severe asthma. In terms of effectiveness, results from the meta-analyses confirmed that CDM programmes were significantly favourable to patients. Indeed, they improved asthma-specific QoL, asthma severity scores and lung function across all designs, with the size of effect varying by study design: CBAs and BAs showing more often larger effects than RCTs. CDM interventions also significantly improved self-efficacy scores in CBAs and BAs, while the meta-analysis was inconclusive in RCTs. In addition, we were able to perform meta-analysis of healthcare use in this augmented review, which was not possible in the Cochrane review: while RCTs did not show significant improvement for healthcare use outcomes, BAs showed lower likelihood of hospitalisations and unscheduled visits after CDM interventions. Finally, the augmented review permitted the consideration of additional outcomes: CDM interventions were associated with decreased likelihood of patients missing days off work or school in BAs, and increased likelihood of using an action plan in BAs, CBAs, and one XS.

Adding NRS and NCS to the original Cochrane review increased the applicability of the Cochrane evidence, as the variety of settings and profile of patients in the newly added studies represent probably better the general setting and target patient population of most asthma CDM programmes. Previous studies have already reported that patient populations selected for recruitment in RCTs in the respiratory field, and more specifically in asthma, represent only about 5 % of the patient population being treated by clinicians in everyday practice [90]. Although RCTs are important and necessary to establish the efficacy of new therapies and interventions, their usefulness can be limited when evaluating how complex interventions will perform in routine care in the real world [91, 92]. In fact, new guidance on conducting systematic reviews of effectiveness interventions encourages the inclusion of non-RCTs to allow for in-depth descriptions of study components and the context and process of implementing the intervention [93]. A few years after that publication, a meta-review of study design criteria in systematic reviews of health systems interventions positively showed that only one third of reviews restricted study designs to RCTs [94].

Overall, evidence from all study designs was consistent in terms of the direction of the effect; because of the increased applicability, such results strengthen the conclusion of the Cochrane review that CDM programmes have a positive effect on quality of life and asthma severity, meaningful outcomes for the everyday life of patients with asthma. Adding non-randomised studies also allowed us to run new meta-analyses showing improvements associated with CDM programmes for five outcomes (hospitalisations, unscheduled visits, days off work, self-efficacy and action plan) that could not be performed in the Cochrane review. Despite the increased risk of bias, the results, which align with the hypothesized trends, suggest evidence of reduction of hospitalisations, unscheduled visits and days off work. Such results are encouraging in the context of healthcare spending reductions. According to Schünemann and colleagues’ terminology [95], the NRS and NCS evidence can be considered as complementary (i.e. NRS and NCS provide additional information on whether the intervention works in different populations) and sequential (i.e. information not (yet) obtained or available from RCTs).

When looking at the effect sizes of RCT, NRS and NCS, the proportion of outcomes reported by authors as improved in response to CDM was higher in the newly added studies compared with the studies in the Cochrane systematic review. The newly added studies (NRS and NCS) also showed both larger effect size than RCTs and larger confidence intervals, indicating uncertainty for the results in addition to high statistical heterogeneity. These results fuel the ongoing debate about the impact of study design and associated biases on study effect size. A number of reviews have been conducted comparing effect sizes and biases of RCTs with non-randomised studies and non-controlled studies. While some reviews found that lack of randomisation or inadequate randomisation was associated with selection bias, larger effects, smaller effects, or reversed direction of effects [14, 19, 96, 97], others found little to no difference in effect sizes between study designs or according to study quality [98, 99]. As suggested by Rockers and colleagues, non-experiments can produce valid and causal effect estimates, in settings that are more natural than RCTs [92]. More research is clearly needed on the issue of study design, risk of bias assessment and effect size. A special emphasis should be put on sound methods for measuring and dealing with confounding bias in NRS studies and other biases in NCS, as well as valid methods for pooling NRS and NCS [100, 101].

Overall, randomised, non-randomised and non-controlled studies included in this augmented review were of moderate to low quality with regards to reporting, statistical and methodology standards. While risk of presenting uncertain results without knowing the direction or magnitude of the effect holds true for both non-randomised and randomised controlled trials, risk of bias and confounding is probably higher in non-randomised trials [97]. However, a study at high risk of bias does not necessarily mean the study results are actually biased. In the case of asthma CMD programmes, other authors conducting similar systematic reviews have reported similar low quality of studies [102, 103]. One reason for this low quality may be the lack of widely accepted methods for evaluating CDM programmes, which are typical complex interventions, built up from a number of interacting components, aiming to modify both providers and patients behaviours, targeting multiple organisational levels and groups, including a large number and variety of outcomes and allowing a certain degree of flexibility (i.e. intervention tailored to patients) [93]. Such complex interventions are difficult to evaluate [104, 105], emphasizing the need for sound evaluation methods to evaluate routine practice in actual settings [106]. Recent guidance has been published to help authors in developing and evaluating complex interventions in general [93, 107] and CDM in particular [106, 108]. Guidelines to improve the completeness and precisions of reporting interventions and their effectiveness have also been published for a variety of study designs, such as the template for intervention description and replication (TIDieR) [109], SQUIRE 2.0 for quality improvement studies [110], the CONSORT statement for RCTs [111], and STROBE for cohort, case–control and cross-sectional studies [112]. Future CDM evaluations should follow these available guidelines to produce high-quality evidence that can then be synthesized in systematic reviews.

The main limitation of this review is that the initial search of the Cochrane review was restricted to quantitative studies, not including all types of available evidence on CDM programmes, such as evidence from implementation, qualitative and mixed methods research which could lead to a more comprehensive understanding of complex interventions.

## Conclusions

Evidence from NRS and NCS complemented RCT evidence from the Cochrane review. In fact, adding NRS and NCS to the original Cochrane review both increased the directness of the evidence, as the variety of settings and profile of patients in the newly added studies probably better represent the general setting and target patient population of typical asthma CDM programmes, and also strengthened the message that CDM programmes have a positive effect on quality of life and asthma severity, which are meaningful outcomes for the everyday life of patients with asthma. The inclusion of study designs other than RCTs in systematic reviews investigating the effectiveness of healthcare interventions, especially when they are complex, is increasingly considered to allow for in-depth descriptions of study components and the context and process of implementing the intervention [93, 94, 97]. However, while NRS and NCS studies provide results from interventions in real-life settings that may be valuable to patients, healthcare providers, managers and policymakers, among others, the methodological quality of the studies remains suboptimal, calling for caution in effectiveness results’ interpretation, as well as for improvements in CDM evaluation methods and reporting by health researchers.

## Additional files

Additional file 1: Risk of bias tool. The extraction form used to assess the risk of bias of included studies. (PDF 55 kb)
Additional file 2: Supplementary Table: Key components of included studies. A table detailing key components of included studies. (PDF 99 kb)

## Abbreviations

BA: Before-after
CBA: Controlled before-after
CDM: Chronic disease management
CI: Confidence interval
EPOC: Effective practice and organisation of care
FEV1: Forced expiratory volume in one second
ICS: Inhaled corticosteroids
ITS: Interrupted time series
MD: Mean difference
NCT: Non-controlled studies
NRS: Non-randomised studies
OR: Odds ratio
PEFR: Peak expiratory flow rate
QoL: Quality of life
QRCT: Quasi-randomised controlled trial
RCT: Randomised controlled trial
SMD: Standardised mean difference
XS: Cross-sectional

## References

[1] Lozano R, Naghavi M, Foreman K, Lim S, Shibuya K, Aboyans V (2012). Global and regional mortality from 235 causes of death for 20 age groups in 1990 and 2010: a systematic analysis for the Global Burden of Disease Study 2010. Lancet.

[2] Levy BD, Noel PJ, Freemer MM, Cloutier MM, Georas SN, Jarjour NN (2015). Future research directions in asthma: An NHLBI Working Group Report. Am J Respir Crit Care Med.

[3] To T, Stanojevic S, Moores G, Gershon AS, Bateman ED, Cruz AA (2012). Global asthma prevalence in adults: findings from the cross-sectional world health survey. BMC Public Health.

[4] Vos T, Flaxman AD, Naghavi M, Lozano R, Michaud C, Ezzati M (2012). Years lived with disability (YLDs) for 1160 sequelae of 289 diseases and injuries 1990–2010: a systematic analysis for the Global Burden of Disease Study 2010. Lancet.

[5] Global Initative for Asthma. Global Strategy for Asthma Management and Prevention. 2015. www.ginasthma.org. Accessed 24 July 2015.

[6] Lemmens KM, Nieboer AP, van Schayck CP, Asin JD, Huijsman R (2008). A model to evaluate quality and effectiveness of disease management. Qual Saf Health Care.

[7] Faxon DP, Schwamm LH, Pasternak RC, Peterson ED, McNeil BJ, Bufalino V (2004). Improving quality of care through disease management: principles and recommendations from the American Heart Association’s Expert Panel on Disease Management. Circulation.

[8] Krumholz HM, Currie PM, Riegel B, Phillips CO, Peterson ED, Smith R (2006). A taxonomy for disease management: a scientific statement from the American Heart Association Disease Management Taxonomy Writing Group. Circulation.

[9] DMAA The Care Continuum Alliance. DMAA Definition of Disease Management. 2010. http://web.archive.org/web/20100815083130/http://www.dmaa.org/dm_definition.asp. Accessed 24 July 2015.

[10] Epping-Jordan JE, Pruitt SD, Bengoa R, Wagner EH (2004). Improving the quality of health care for chronic conditions. Qual Saf Health Care.

[11] Wagner EH, Austin BT, Davis C, Hindmarsh M, Schaefer J, Bonomi A (2001). Improving chronic illness care: translating evidence into action. Health Aff (Millwood).

[12] Peytremann-Bridevaux I, Burnand B (2009). Disease management: a proposal for a new definition. Int J Integr Care.

[13] Bravata DM, McDonald KM, Shojania KG, Sundaram V, Owens DK (2005). Challenges in systematic reviews: synthesis of topics related to the delivery, organization, and financing of health care. Ann Intern Med.

[14] Deeks JJ, Dinnes J, D’Amico R, Sowden AJ, Sakarovitch C, Song F (2003). Evaluating non-randomised intervention studies. Health Technol Assess.

[15] Davidoff F, Batalden P, Stevens D, Ogrinc G, Mooney S, Group SD (2008). Publication guidelines for improvement studies in health care: evolution of the SQUIRE project. Ann Intern Med.

[16] Conklin A, Nolte E (2011). Disease management evaluation: a comprehensive review of current state of the art.

[17] Tinetti ME, Studenski SA (2011). Comparative effectiveness research and patients with multiple chronic conditions. N Engl J Med.

[18] Norris SL, Atkins D (2005). Challenges in using nonrandomized studies in systematic reviews of treatment interventions. Ann Intern Med.

[19] Anglemyer A, Horvath HT, Bero L (2014). Healthcare outcomes assessed with observational study designs compared with those assessed in randomized trials. Cochrane Database Syst Rev.

[20] Peytremann-Bridevaux I, Arditi C, Gex G, Bridevaux PO, Burnand B (2015). Chronic disease management programmes for adults with asthma. Cochrane Database Syst Rev.

[21] Effective Practice and Organisation of Care (EPOC) (2013). What study designs should be included in an EPOC review?. EPOC Resources for review authors.

[22] Effective Practice and Organisation of Care (EPOC) (2015). Suggested risk of bias criteria for EPOC reviews. EPOC Resources for review authors.

[23] Sterne JAC, Higgins JPT, Reeves BC on behalf of the development group for ACROBAT-NRSI. A Cochrane Risk Of Bias Assessment Tool: for Non-Randomized Studies of Interventions (ACROBAT-NRSI), Version 1.0.0. 24 Sept 2014. www.riskofbias.info. Accessed 23 Sept 2015.

[24] Downs SH, Black N (1998). The feasibility of creating a checklist for the assessment of the methodological quality both of randomised and non-randomised studies of health care interventions. J Epidemiol Community Health.

[25] Thomas BH, Ciliska D, Dobbins M, Micucci S (2004). A process for systematically reviewing the literature: providing the research evidence for public health nursing interventions. Worldviews Evid Based Nurs.

[26] Armijo-Olivo S, Stiles CR, Hagen NA, Biondo PD, Cummings GG (2012). Assessment of study quality for systematic reviews: a comparison of the Cochrane Collaboration Risk of Bias Tool and the Effective Public Health Practice Project Quality Assessment Tool: methodological research. J Eval Clin Pract.

[27] Valentine JC, Thompson SG (2013). Issues relating to confounding and meta-analysis when including non-randomized studies in systematic reviews on the effects of interventions. Res Synth Methods.

[28] Higgins JPT, Green S, editors. Cochrane Handbook for Systematic Reviews of Interventions. Version 5.1.0 [updated March 2011]. The Cochrane Collaboration. 2011. http://handbook.cochrane.org. Accessed 24 July 2015.

[29] Effective Practice and Organisation of Care (EPOC) (2013). Interrupted time series (ITS) analyses. EPOC resources for review authors.

[30] Review Manager (RevMan) [Computer program]. Version 5.3. Copenhagen: The Nordic Cochrane Centre. The Cochrane Collaboration; 2014. http://community.cochrane.org/tools/review-production-tools/revman-5/about.

[31] Allen-Ramey FC, Diette GB, McDonald RC, Skinner EA, Steinwachs DM, Wu AW (2002). Methods aimed at improving asthma care and outcomes management: a case study. Dis Manag Health Out.

[32] Curtin K, Hayes BD, Holland CL, Katz LA (1998). Computer-generated intervention for asthma population care management. Eff Clin Pract.

[33] Gaude GS, Hattiholi J, Chaudhury A (2014). Role of health education and self-action plan in improving the drug compliance in bronchial asthma. J Fam Med Prim Care.

[34] Kelso TM, Abou-Shala N, Heilker GM, Arheart KL, Portner TS, Self TH (1996). Comprehensive long-term management program for asthma: effect on outcomes in adult African-Americans. Am J Med Sci.

[35] Saini B, Filipovska J, Bosnic-Anticevich S, Taylor S, Krass I, Armour C (2008). An evaluation of a community pharmacy-based rural asthma management service. Aust J Rural Health.

[36] Saini B, Krass I, Armour C (2004). Development, implementation, and evaluation of a community pharmacy-based asthma care model. Ann Pharmacother.

[37] Tatis V, Remache D, DiMango E (2005). Results of a culturally directed asthma intervention program in an inner-city Latino community. Chest.

[38] Brandao HV, Cruz CM, Santos Ida S, Ponte EV, Guimaraes A, Augusto Filho A (2009). Hospitalizations for asthma: impact of a program for the control of asthma and allergic rhinitis in Feira de Santana, Brazil. J Bras Pneumol.

[39] Charlton I, Charlton G, Broomfield J, Campbell M (1992). An evaluation of a nurse-run asthma clinic in general practice using an attitudes and morbidity questionnaire. Fam Pract.

[40] Charlton I, Charlton G, Broomfield J, Mullee MA (1991). Audit of the effect of a nurse run asthma clinic on workload and patient morbidity in a general practice. Br J Gen Pract.

[41] D’Souza W, Burgess C, Ayson M, Crane J, Pearce N, Beasley R (1996). Trial of a “credit card” asthma self-management plan in a high-risk group of patients with asthma. J Allergy Clin Immunol.

[42] D’Souza W, Crane J, Burgess C, Te Karu H, Fox C, Harper M (1994). Community-based asthma care: trial of a “credit card” asthma self-management plan. Eur Respir J.

[43] Emmerton L, Shaw J, Kheir N (2003). Asthma management by New Zealand pharmacists: a pharmaceutical care demonstration project. J Clin Pharm Ther.

[44] Franco R, Nascimento HF, Cruz AA, Santos AC, Souza-Machado C, Ponte EV (2009). The economic impact of severe asthma to low-income families. Allergy.

[45] Franco R, Santos AC, do Nascimento HF, Souza-Machado C, Ponte E, Souza-Machado A (2007). Cost-effectiveness analysis of a state funded programme for control of severe asthma. BMC Public Health.

[46] Hertzman PA, Kelly HW, Coultas D (2005). Chronic illness care in Russia: a pilot project to improve asthma care in a “closed city”. Chest.

[47] Ignacio-Garcia JM, Pinto-Tenorio M, Chocron-Giraldez MJ, Cabello-Rueda F, Lopez-Cozar Gil AI, Ignacio-Garcia JM (2002). Benefits at 3 yrs of an asthma education programme coupled with regular reinforcement. Eur Respir J.

[48] Licskai C, Sands T, Ong M, Paolatto L, Nicoletti I (2012). Using a knowledge translation framework to implement asthma clinical practice guidelines in primary care. Int J Qual Health Care.

[49] Lind A, Kaplan L, Berg GD (2006). Evaluation of an asthma disease management program in a Medicaid population. Dis Manag Health Out.

[50] Lindberg M, Ahlner J, Moller M, Ekstrom T (1999). Asthma nurse practice--a resource-effective approach in asthma management. Respir Med.

[51] Maljanian R, Wolf S, Goethe J, Hernandez P, Horowitz S (1999). An inner-city asthma disease management initiative: results of an outcomes evaluation. Dis Manag Health Out.

[52] Mangiapane S, Schulz M, Muhlig S, Ihle P, Schubert I, Waldmann HC (2005). Community pharmacy-based pharmaceutical care for asthma patients. Ann Pharmacother.

[53] Narhi U, Airaksinen M, Tanskanen P, Enlund H (2001). The effects of a pharmacy-based intervention on the knowledge and attitudes of asthma patients. Patient Educ Couns.

[54] Patel PH, Welsh C, Foggs MB (2004). Improved asthma outcomes using a coordinated care approach in a large medical group. Dis Manag.

[55] Pauley TR, Magee MJ, Cury JD (1995). Pharmacist-managed, physician-directed asthma management program reduces emergency department visits. Ann Pharmacother.

[56] Ponte E, Franco RA, Souza-Machado A, Souza-Machado C, Cruz AA (2007). Impact that a program to control severe asthma has on the use of Unified Health System resources in Brazil. J Bras Pneumol.

[57] Steuten L, Vrijhoef B, Van Merode F, Wesseling GJ, Spreeuwenberg C (2006). Evaluation of a regional disease management programme for patients with asthma or chronic obstructive pulmonary disease. Int J Qual Health Care.

[58] To T, Cicutto L, Degani N, McLimont S, Beyene J (2008). Can a community evidence-based asthma care program improve clinical outcomes? A longitudinal study. Med Care.

[59] Tschopp JM, Frey JG, Pernet R, Burrus C, Jordan B, Morin A (2002). Bronchial asthma and self-management education: implementation of guidelines by an interdisciplinary programme in health network. Swiss Med Wkly.

[60] Boonsawat W, Eiadprapan W (2013). Successful of the National Asthma Program using the Easy Asthma Clinic Model to improve Asthma Management in Thailand. Respirology.

[61] Fireman B, Bartlett J, Selby J (2004). Can disease management reduce health care costs by improving quality?. Health Aff (Millwood).

[62] Haahtela T, Tuomisto LE, Pietinalho A, Klaukka T, Erhola M, Kaila M (2006). A 10 year asthma programme in Finland: major change for the better. Thorax.

[63] Kauppi P, Linna M, Martikainen J, Makela MJ, Haahtela T (2013). Follow-up of the Finnish Asthma Programme 2000-2010: reduction of hospital burden needs risk group rethinking. Thorax.

[64] Mehring M, Donnachie E, Mutschler R, Hofmann F, Keller M, Schneider A (2013). Disease management programs for patients with asthma in Germany: a longitudinal population-based study. Respir Care.

[65] Souza-Machado C, Souza-Machado A, Franco R, Ponte EV, Barreto ML, Rodrigues LC (2010). Rapid reduction in hospitalisations after an intervention to manage severe asthma. Eur Respir J.

[66] Carmo TA, Andrade SM, Cerci Neto A (2011). Evaluation of an asthma control program in family health units. Cad Saude Publica.

[67] Lindberg M, Ahlner J, Ekstrom T, Jonsson D, Moller M (2002). Asthma nurse practice improves outcomes and reduces costs in primary health care. Scand J Caring Sci.

[68] Armour C, Bosnic-Anticevich S, Brillant M, Burton D, Emmerton L, Krass I (2007). Pharmacy Asthma Care Program (PACP) improves outcomes for patients in the community. Thorax.

[69] Cambach W, Chadwick-Straver RV, Wagenaar RC, van Keimpema AR, Kemper HC (1997). The effects of a community-based pulmonary rehabilitation programme on exercise tolerance and quality of life: a randomized controlled trial. Eur Respir J.

[70] Castro M, Zimmermann NA, Crocker S, Bradley J, Leven C, Schechtman KB (2003). Asthma intervention program prevents readmissions in high healthcare users. Am J Respir Crit Care Med.

[71] Charrois TL, Newman SC, Senthilselvan A, Tsuyuki RT (2006). Improving asthma control in the rural setting: The BREATHE (Better Respiratory Education and Asthma Treatment in Hinton and Edson) study. Can Pharm J (Ott).

[72] Couturaud F, Proust A, Frachon I, Dewitte JD, Oger E, Quiot JJ (2002). Education and self-management: a one-year randomized trial in stable adult asthmatic patients. J Asthma.

[73] Galbreath AD, Smith B, Wood PR, Inscore S, Forkner E, Vazquez M (2008). Assessing the value of disease management: Impact of 2 disease management strategies in an underserved asthma population. Ann Allergy Asthma Immunol.

[74] Huang TT, Li YT, Wang CH (2009). Individualized programme to promote self-care among older adults with asthma: randomized controlled trial. J Adv Nurs.

[75] Kokubu F, Nakajima S, Ito K, Makino S, Kitamura S, Fukuchi Y (2000). Hospitalization reduction by an asthma tele-medicine system. Arerugi.

[76] Martin MA, Catrambone CD, Kee RA, Evans AT, Sharp LK, Lyttle C (2009). Improving asthma self-efficacy: developing and testing a pilot community-based asthma intervention for African American adults. J Allergy Clin Immunol.

[77] Mayo PH, Richman J, Harris HW (1990). Results of a program to reduce admissions for adult asthma [see comment]. Ann Intern Med.

[78] McLean W, Gillis J, Waller R (2003). The BC Community Pharmacy Asthma Study: A study of clinical, economic and holistic outcomes influenced by an asthma care protocol provided by specially trained community pharmacists in British Columbia. Can Respir J.

[79] Petro W, Schulenburg JM, Greiner W, Weithase J, Schulke A, Metzdorf N (2005). Efficacy of a disease management programme in asthma. Pneumologie.

[80] Schatz M, Gibbons C, Nelle C, Harden K, Zeiger RS (2006). Impact of a care manager on the outcomes of higher risk asthmatic patients: a randomized controlled trial. J Asthma.

[81] Smith JR, Mildenhall S, Noble MJ, Shepstone L, Koutantji M, Mugford M (2005). The Coping with Asthma Study: a randomised controlled trial of a home based, nurse led psychoeducational intervention for adults at risk of adverse asthma outcomes. Thorax.

[82] Wilson SR, Strub P, Buist AS, Knowles SB, Lavori PW, Lapidus J (2010). Shared treatment decision making improves adherence and outcomes in poorly controlled asthma. Am J Respir Crit Care Med.

[83] Herborg H, Soendergaard B, Froekjaer B, Fonnesbaek L, Jorgensen T, Hepler CD (2001). Improving drug therapy for patients with asthma--part 1: Patient outcomes. J Am Pharm Assoc (Wash).

[84] Herborg H, Soendergaard B, Jorgensen T, Fonnesbaek L, Hepler CD, Holst H (2001). Improving drug therapy for patients with asthma-part 2: Use of antiasthma medications. J Am Pharm Assoc (Wash).

[85] Feifer RA, Verbrugge RR, Khalid M, Levin R, O’Keefe GB, Aubert RE (2004). Improvements in asthma pharmacotherapy and self-management: an example of a population-based disease management program. Dis Manag Health Out.

[86] Landon BE, Hicks LS, O’Malley AJ, Lieu TA, Keegan T, McNeil BJ (2007). Improving the management of chronic disease at community health centers. N Engl J Med.

[87] Weng HC (2005). Impacts of a government-sponsored outpatient-based disease management program for patients with asthma: a preliminary analysis of national data from Taiwan. Dis Manag.

[88] Windt R, Glaeske G (2010). Effects of a German asthma disease management program using sickness fund claims data. J Asthma.

[89] Guyatt GH, Oxman AD, Vist GE, Kunz R, Falck-Ytter Y, Alonso-Coello P (2008). GRADE: an emerging consensus on rating quality of evidence and strength of recommendations. BMJ.

[90] Herland K, Akselsen J-P, Skjønsberg OH, Bjermer L (2005). How representative are clinical study patients with asthma or COPD for a larger “real life” population of patients with obstructive lung disease?. Respir Med.

[91] Price D, Brusselle G, Roche N, Freeman D, Chisholm A (2015). Real-world research and its importance in respiratory medicine. Breathe.

[92] Rockers PC, Rottingen JA, Shemilt I, Tugwell P, Barnighausen T (2015). Inclusion of quasi-experimental studies in systematic reviews of health systems research. Health Policy.

[93] Craig P, Dieppe P, Macintyre S, Michie S, Nazareth I, Petticrew M (2008). Developing and evaluating complex interventions: the new Medical Research Council guidance. BMJ.

[94] Rockers PC, Feigl AB, Rottingen JA, Fretheim A, de Ferranti D, Lavis JN (2012). Study-design selection criteria in systematic reviews of effectiveness of health systems interventions and reforms: A meta-review. Health Policy.

[95] Schünemann HJ, Tugwell P, Reeves BC, Akl EA, Santesso N, Spencer FA (2013). Non-randomized studies as a source of complementary, sequential or replacement evidence for randomized controlled trials in systematic reviews on the effects of interventions. Res Synth Methods.

[96] Kunz R, Oxman AD (1998). The unpredictability paradox: review of empirical comparisons of randomised and non-randomised clinical trials. BMJ.

[97] Peinemann F, Tushabe DA, Kleijnen J (2013). Using multiple types of studies in systematic reviews of health care interventions--a systematic review. PLoS One.

[98] MacLehose RR, Reeves BC, Harvey IM, Sheldon TA, Russell IT, Black AM (2000). A systematic review of comparisons of effect sizes derived from randomised and non-randomised studies. Health Technol Assess.

[99] Oliver S, Bagnall AM, Thomas J, Shepherd J, Sowden A, White I (2010). Randomised controlled trials for policy interventions: a review of reviews and meta-regression. Health Technol Assess.

[100] Reeves BC, Higgins JPT, Ramsay C, Shea B, Tugwell P, Wells GA (2013). An introduction to methodological issues when including non-randomised studies in systematic reviews on the effects of interventions. Res Synth Methods.

[101] Methodology Committee of the Patient-Centered Outcomes Research I (2012). Methodological standards and patient-centeredness in comparative effectiveness research: the PCORI perspective. JAMA.

[102] Maciejewski ML, Chen SY, Au DH (2009). Adult asthma disease management: an analysis of studies, approaches, outcomes, and methods. Respir Care.

[103] Lemmens KM, Nieboer AP, Huijsman R (2009). A systematic review of integrated use of disease-management interventions in asthma and COPD. Respir Med.

[104] Campbell M, Fitzpatrick R, Haines A, Kinmonth AL, Sandercock P, Spiegelhalter D (2000). Framework for design and evaluation of complex interventions to improve health. BMJ.

[105] Campbell NC, Murray E, Darbyshire J, Emery J, Farmer A, Griffiths F (2007). Designing and evaluating complex interventions to improve health care. BMJ.

[106] Nolte E, Conklin A, Adams JL, Brunn M, Cadier B, Chevreul K (2012). Evaluating chronic disease management. Recommendations for funders and users.

[107] Mohler R, Kopke S, Meyer G (2015). Criteria for reporting the development and evaluation of complex interventions in healthcare: revised guideline (CReDECI 2). Trials.

[108] Linden A, Adams JL, Roberts N (2013). Evaluation methods in disease management: determining program effectiveness.

[109] Hoffmann TC, Glasziou PP, Boutron I, Milne R, Perera R, Moher D (2014). Better reporting of interventions: template for intervention description and replication (TIDieR) checklist and guide. BMJ.

[110] Ogrinc G, Davies L, Goodman D, Batalden P, Davidoff F, Stevens D (2015). SQUIRE 2.0 (Standards for QUality Improvement Reporting Excellence): revised publication guidelines from a detailed consensus process. BMJ Qual Saf.

[111] Moher D, Hopewell S, Schulz KF, Montori V, Gotzsche PC, Devereaux PJ (2010). CONSORT 2010 explanation and elaboration: updated guidelines for reporting parallel group randomised trials. BMJ.

[112] von Elm E, Altman DG, Egger M, Pocock SJ, Gotzsche PC, Vandenbroucke JP (2007). The Strengthening the Reporting of Observational Studies in Epidemiology (STROBE) statement: guidelines for reporting observational studies. Epidemiology.

