# Supplementary material for: Adding non-randomised studies to a Cochrane review brings complementary information for healthcare stakeholders: an augmented systematic review and meta-analysis
Source: BMC Health Serv Res. 2016 Oct 21;16:598. doi: 10.1186/s12913-016-1816-5 (PMC5073845; doi:10.1186/s12913-016-1816-5)
Supplement: Additional file 1: — Risk of bias tool. The extraction form used to assess the risk of bias of included studies. (PDF 55 kb) [file 12913_2016_1816_MOESM1_ESM.pdf]

Adding NRS to a Cochrane review on CDM and asthma: an augmented systematic review and meta-analysis / Risk of bias extraction form

DOMAIN	DESIGN	INSTRUCTIONS	REVIEW AUTHORS' JUDGEMENT
Selection bias			
**Random sequence generation**<br>(COCHRANE, EPOC)	RCT<br>QRCT<br>CBA	Score YES if a random component in the sequence generation process is described (e.g. referring to a random number table). Score NO when a nonrandom method is used (e.g. performed by date of admission). NRCTs and CBA studies should be scored NO. Score UNCLEAR if not specified in the paper.	YES (Low risk bias) □ NO (High risk bias) □ UNCLEAR □<br>Quote/comment :
**Allocation concealment**<br>(COCHRANE, EPOC)	RCT<br>QRCT<br>CBA	Score YES if the unit of allocation was by institution, team or professional and allocation was performed on all units at the start of the study; or if the unit of allocation was by patient or episode of care and there was some form of centralised randomisation scheme, an on-site computer system or sealed opaque envelopes were used. CBA studies should be scored NO. Score UNCLEAR if not specified in the paper.	YES (Low risk bias) □ NO (High risk bias) □ UNCLEAR □<br>Quote/comment :
**Baseline characteristics similar**<br>(EPOC)	RCT<br>QRCT<br>CBA	Score YES if baseline characteristics of the study and control patients are reported and similar. Score UNCLEAR if it is not clear in the paper (e.g. characteristics are mentioned in text but no data were presented). Score NO if there is no report of characteristics in text or tables or if there are differences between control and intervention patients. Note that in some cases imbalance in patient characteristics may be due to recruitment bias whereby the provider was responsible for recruiting patients into the trial.	YES (Low risk bias) □ NO (High risk bias) □ UNCLEAR □<br>Quote/comment :
**Baseline outcomes similar**<br>(EPOC)	RCT<br>QRCT<br>CBA	Score YES if performance or patient outcomes were measured prior to the intervention, and no important differences were present across study groups. In RCTs, score YES if imbalanced but appropriate adjusted analysis was performed (e.g. Analysis of covariance). Score NO if important differences were present and not adjusted for in analysis. If RCTs have no baseline measure of outcome, score UNCLEAR.	YES (Low risk bias) □ NO (High risk bias) □ UNCLEAR □<br>Quote/comment :
**Confounding unlikely**<br>(ACROBAT-NRSI, DOWNS)	all	General judgment about confounding risk. Score NO for all non-controlled studies and NO if: the main conclusions of the study were based on analyses of treatment rather than intention to treat; or the distribution of known confounders differed between the treatment groups but was not taken into account in the analyses. In non-randomised studies if the effect of the main confounders was not investigated or confounding was demonstrated but no adjustment was made in the final analyses the question should be answered as NO. Score UNCLEAR if the distribution of known confounders in the different treatment groups was not described.	YES (Low risk bias) □ NO (High risk bias) □ UNCLEAR □<br>Quote/comment :

**Appropriate analyses**<br><br>(DOWNS, EPHPP)	all	Did the authors use an appropriate analysis method that adjusted for all the critically important confounding domains (e.g. stratification, regression, matching, standardization, propensity score) for RCT, QRCT, CBA and/or adjusted for clusters if cluster design and/or use trend analysis for ITS?	YES (Low risk bias) □ NO (High risk bias) □ UNCLEAR □<br>Quote/comment :
**Sample representative of source population**<br><br>(DOWNS, EPHPP)	BA<br>ITS<br>XS	The study must identify the source population for patients and describe how the patients were selected. Score YES if patients comprised the entire source population, an unselected sample of consecutive patients, or a random sample. Random sampling is only feasible where a list of all members of the relevant population exists. Score UNCLEAR if a study does not report the proportion of the source population from which the patients are derived.	YES (Low risk bias) □ NO (High risk bias) □ UNCLEAR □<br>Quote/comment :
**Intervention independent of other changes**<br><br>(EPOC)	BA<br>ITS<br>XS	Score YES if there are compelling arguments that the intervention occurred independently of other changes over time and the outcome was not influenced by other confounding variables/historic events during study period. *If Events/variables identified, note what they are.* Score NO if reported that intervention was not independent of other changes in time.	YES (Low risk bias) □ NO (High risk bias) □ UNCLEAR □<br>Quote/comment :
Performance bias			
**Intervention integrity**<br><br>(ACROBAT-NRSI, DOWNS, EPOC, EPHPP)	all	Score YES if allocation was by community, institution or practice and it is unlikely that the control group received the intervention. Also score YES if numbers of switches to other interventions was low and if implementation failure was minor. If not, were adjustments techniques used that are likely to correct for these issues? If yes, then score YES. If not, then score NO. Score NO if it is likely that the control group received the intervention (e.g. if patients rather than professionals were randomized). Score UNCLEAR if professionals were allocated within a clinic or practice and it is possible that communication between intervention and control professionals could have occurred (e.g. physicians within practices were allocated to intervention or control)	YES (Low risk bias) □ NO (High risk bias) □ UNCLEAR □<br>Quote/comment :
Detection bias			
**Blinding of outcome assessment**<br><br>(COCHRANE, EPOC)	all	Score YES if the authors state explicitly that the primary outcome variables were assessed blindly, or the outcomes are objective, e.g. length of hospital stay. Score NO if the outcomes were not assessed blindly and outcome measurement is likely to be influenced by lack of blinding. Score UNCLEAR if not specified in the paper.	YES (Low risk bias) □ NO (High risk bias) □ UNCLEAR □<br>Quote/comment :

Attrition bias			
**Incomplete outcome data addressed**<br><br>(COCHRANE, EPOC)	all	Score YES if no missing data or missing outcome measures were unlikely to bias the results (e.g. the proportion of missing data was similar in the intervention and control groups or the proportion of missing data was less than the effect size i.e. unlikely to overturn the study result). Score NO if missing outcome data was likely to bias the results. Score UNCLEAR if not specified in the paper (Do not assume 100% follow up unless stated explicitly).	YES (Low risk bias) □ NO (High risk bias) □ UNCLEAR □<br><br>Quote/comment :
Other bias			
**Free of other bias**<br><br>(COCHRANE, EPOC)<br><br>e.g. cluster-RCT: recruitment bias	all	Score YES if study appears to be free of other sources of bias. Score NO if there is at least one important risk of bias (e.g. study has been claimed to have been fraudulent, had some other problem). Score UNCLEAR if there is insufficient information to assess whether an important risk of bias exists.	YES (Low risk bias) □ NO (High risk bias) □ UNCLEAR □<br><br>Quote/comment :

We indicated in brackets which tool(s) the domain was extracted from.
ACROBAT-NRSI: A Cochrane Risk Of Bias Assessment Tool for Non- Randomized Studies of Interventions. www.riskofbias.info [accessed 23 September 2015]
COCHRANE: The Cochrane Collaboration's tool for assessing risk of Bias. http://handbook.cochrane.org/chapter_8/table_8_5_a_the_cochrane_collaborations_tool_for_assessing.htm [accessed 24 July 2015]
DOWNS: The feasibility of creating a checklist for the assessment of the methodological quality both of randomised and non-randomised studies of health care interventions. http://www.ncbi.nlm.nih.gov/pmc/articles/PMC1756728/pdf/v052p00377.pdf [accessed 24 July 2015]
EPOC: Suggested risk of bias criteria for EPOC reviews. http://epoc.cochrane.org/sites/epoc.cochrane.org/files/uploads/14%20Suggested%20risk%20of%20bias%20criteria%20for%20EPOC%20reviews%202015%2009%2002.pdf [accessed 24 July 2015]
EPHPP: Quality assessment tool for quantitative studies, Effective Public Health Practice Project. A generic tool used to evaluate a variety of intervention study designs such as RCTs, before-and-after and case-control studies. http://www.ephpp.ca/tools.html [accessed 24 July 2015]
