# Supplementary material for: Adding non-randomised studies to a Cochrane review brings complementary information for healthcare stakeholders: an augmented systematic review and meta-analysis
Source: BMC Health Serv Res. 2016 Oct 21;16:598. doi: 10.1186/s12913-016-1816-5 (PMC5073845; doi:10.1186/s12913-016-1816-5)
Supplement: Additional file 2: — Supplementary Table: Key components of included studies. A table detailing key components of included studies. (PDF 99 kb) [file 12913_2016_1816_MOESM2_ESM.pdf]

**Adding NRS to a Cochrane review on CDM and asthma: an augmented systematic review and meta-analysis / Supplementary Table: Key components of included studies**

| Study ID | Design (allocation) | Country HC setting | Patients | Intervention (name[1], duration, number of components, main component) | Comparison | Outcomes |
|---|---|---|---|---|---|---|
| Armour 2007 | C-RCT (pharmacy) | Australia<br>Rural and urban pharmacies | N=346<br>women: 63.7.%<br>mean age: 49.1<br>asthma: mod/sev | Pharmacy Asthma Care Program<br>6 months<br>components: 8<br>main: EDU | Usual care (including minimal training for pharmacists) | Org: 1<br>Process: 6<br>QOL: 1<br>Symptoms/activity: 2<br>Self-care: 5<br>Lung function: 2 |
| Cambach 1997 | RCT | Netherlands<br>Local physiotherapy practices | N=43<br>women: 74.4%<br>mean age: 46.3<br>asthma: mild/mod | Rehabilitation program<br>3 months<br>components: 6<br>main: ORG_PT | Usual care | QOL: 1<br>Symptoms/activity: 3 |
| Castro 2003 | RCT | USA<br>Inpatients and outpatients in a hospital | N=96<br>women: 82.4%<br>mean age: 36.4<br>asthma: mod/sev | Asthma nurse specialist for "high-risk" inpatients<br>6 months<br>components: 10<br>main: ORG_PT / ORG_HC | Usual care (including minimal intervention for patients) | HC use: 8<br>QOL: 1 |
| Charrois 2006 | RCT | Canada<br>Community rural pharmacies and PCPs | N=70<br>women: 53%<br>mean age: 37.2<br>asthma: mod/sev | Better Respiratory Education and Asthma Treatment in Hinton and Edson (BREATHE)<br>6 months<br>components: 11<br>main: EDU / ORG_HC | Usual care (including minimal intervention for patients) | Org: 1<br>Process: 3<br>HC use: 1<br>Symptoms/activity: 2<br>Lung function: 1 |
| Couturaud 2002 | RCT | France<br>Outpatient clinic of two university hospitals | N=54<br>women: 68%<br>mean age: 38<br>asthma: mod/sev | Educational program following treatment readjustment<br>12 months<br>components: 7<br>main: EDU | Usual care | HC use: 1<br>QOL:1<br>Symptoms/activity: 3<br>Self-care: 3<br>Lung function: 1 |
| Galbreath 2008 | RCT | USA<br>University medical centre and a PCP | N=386<br>women: 79%<br>mean age: 42.8<br>asthma: mod/sev | The South Texas Asthma Management Project (STAMP)<br>6 months<br>components: 9<br>main: EDU / ORG_PT | Usual care | Org: 1<br>Process: 2<br>HC use: 4<br>QOL: 1<br>Symptoms/activity: 2<br>Lung function: 3 |
| Huang 2009 | RCT | Taiwan<br>Outpatient chest department of a hospital | N=148<br>women: 27.3%<br>mean age: na<br>asthma: mod/sev | Individualised self-care education program<br>6 months<br>components: 6<br>main: EDU | Usual care (including minimal intervention for patients) | Process: 1<br>HC use: 1<br>Symptoms/activity: 2<br>Self-care: 3<br>Lung function: 4 |
| Kokubu 2000 | RCT | Japan<br>Hospital and patients' home | N=66<br>women: 58.9%<br>mean age: 48.6<br>asthma: na | Asthma telemedicine system<br>6 months<br>components: 8<br>main: ORG_HC | Usual care | HC use: 4<br>Pt satis: 1<br>QOL: 1<br>Symptoms/activity: 3<br>Self-care: 3<br>Lung function: 1 |
| Martin 2009 | RCT | USA<br>PCPs | N=37<br>women: 68.3%<br>mean age: 35<br>asthma:na | Community-based intervention to improve asthma self-efficacy in African American adults designed by the Chicago Initiative to Raise Asthma Health Equity (CHIRAH)<br>3 months<br>components: 7<br>main: EDU | Usual care (including minimal intervention for patients) | Org: 1<br>Process: 2<br>QOL: 1<br>Symptoms/activity: 3<br>Self-care: 3 |
| Mayo 1990 | RCT | USA<br>Outpatient chest clinic of a hospital | N=104<br>women: 63.5%<br>mean age: 42<br>asthma: mod/sev | Outpatient program to reduce readmissions for asthma exacerbations<br>8 months<br>components: 6<br>main: EDU | Usual care | HC use: 1<br>Symptoms/activity: 1 |

| Study ID | Design (allocation) | Country HC setting | Patients | Intervention (name[1], duration, number of components, main component) | Comparison | Outcomes |
|---|---|---|---|---|---|---|
| McLean 2003 | RCT | Canada<br>Community pharmacies | N=224<br>women: 63%<br>mean age: na<br>asthma: na | The British Columbia pharmacy asthma study incorporating an asthma care protocol provided by specially trained community pharmacists<br>12 months<br>components: 7<br>main: EDU | Usual care | HC use: 4<br>Pt satis: 1<br>QOL: 1<br>Symptoms/activity: 4<br>Self-care: 1<br>Lung function: 1 |
| Petro 2005 | C-RCT (provider) | Germany<br>PCPs | N=111<br>women: 49.1%<br>mean age: 56.2<br>asthma: mod/sev | A disease management program with a case manager<br>12 months<br>components: 7<br>main: ORG_HC | Usual care | HC use: 2<br>QOL: 3<br>Symptoms/activity: 1<br>Lung function: 2 |
| Schatz 2006 | RCT | USA<br>Kaiser Permanente Medical Care program | N=45<br>women: 39.8%<br>mean age: 45.1<br>asthma: mod/sev | Regular care manager and intensive individualised educational visit<br>12 months<br>components: 11<br>main: EDU / ORG_PT | Usual care (including minimal intervention for patients) | Process: 1<br>HC use: 1<br>QOL: 1<br>Symptoms/activity: 2<br>Self-care: 1 |
| Smith 2005 | RCT | UK<br>Outpatient asthma clinics of a hospital and PCPs | N=84<br>women: 73%<br>mean age: 36.4<br>asthma: mod/sev | The Coping with Asthma Study (a home based, nurse led psycho-educational intervention for adults at risk of adverse asthma outcomes)<br>6 months<br>components: 15<br>main: EDU | Usual care | QOL: 6<br>Symptoms/activity: 1<br>Self-care: 6 |
| Wilson 2010 | RCT | USA<br>Kaiser Permanente clinics | N=551<br>women: 56.5%<br>mean age: 46.3<br>asthma: mod/sev | The Better Outcomes of Asthma Treatment (BOAT) study<br>9 months<br>components: 9<br>main: EDU / ORG_PT | Usual care (including minimal intervention for patients) | Process: 1<br>HC use: 2<br>QOL: 1<br>Symptoms/activity: 5<br>Lung function: 2 |
| Herborg 2001 | C-QRCT (pharmacy) | Denmark<br>Community pharmacies | N=413<br>women: 56.2%<br>mean age: 40.6<br>asthma: mod/sev | Therapeutic outcomes monitoring (TOM) program<br>12 months<br>components: 9<br>main: ORG_PT | Usual care | Org : 3<br>HC use: 7<br>Pt satis: 1<br>QOL: 2<br>Symptoms/activity: 4<br>Self-care: 1<br>Lung function: 1 |
| Feifer 2004 | CBA* | USA<br>PCPs in a region covered by a health insurance company | N=70,900<br>women: 56%<br>mean age: na<br>asthma:na | Population-based asthma disease management program using broad-based educational interventions<br>12 months<br>components: 7<br>main: EDU / ORG_PT | Usual care | Process: 3<br>HC use: 3<br>QOL: 1<br>Symptoms/activity: 4<br>Self-care: 3 |
| Landon 2000 | CBA* | USA<br>Community health centers | N=3392<br>women: 65.6%<br>mean age: 31.4<br>asthma: na | Health Disparities Collaboratives disseminating quality-improvement techniques<br>54 months<br>components: ≥11<br>main: ORG_HC | Usual care | Process: 7<br>HC use: 1<br>Symptoms/activity: 1 |
| Weng 2005 | CBA* | Taiwan<br>Outpatient clinics of a hospital and PCPs | N=4042<br>women: 43.3%<br>mean age: na<br>asthma: na | A government-sponsored outpatient-based disease management program<br>12 months<br>components: 8<br>main: EDU | Usual care | Pro satis: 1<br>HC use: 4 |
| Windt 2010 | CBA* | Germany<br>PCPs | N=634<br>women: 46.4%<br>mean age: 36.5<br>asthma: na | Nationwide asthma disease management program<br>>12 months<br>components: ≥5<br>main: ORG_HC | Usual care | Process: 8<br>HC use: 2 |
| Allen-Ramey 2002 | CBA | USA<br>Managed care organisation | N=3013<br>women: 70.9%<br>mean age: 46.6<br>asthma: mod/sev | Anthem "Healthy Solutions for Asthma" program<br>3-36 months<br>components: 9<br>main: EDU | Limited intervention for patients and HC professionals | Process: 2<br>HC use: 2<br>Symptoms/activity: 2<br>Self-care: 1 |

| Study ID | Design (allocation) | Country<br>HC setting | Patients | Intervention (name[1], duration, number of components, main component) | Comparison | Outcomes |
|---|---|---|---|---|---|---|
| Curtin 1998 | CBA | USA<br>Health care plan PCPs | N=na<br>women: % na<br>mean age: na<br>asthma: na | Asthma Game Plan and reminder system<br>6 months<br>components: 6<br>main: ORG_HC | Usual care | Process: 2<br>HC use: 3 |
| Gaude 2014 | CBA | India<br>Tertiary care hospital | N=500<br>women: % na<br>mean age: 37<br>asthma: na | Health education and self-action plan to improve the compliance in bronchial asthma patients<br>3 months<br>components: 6<br>main: EDU | Usual care | Self-care: 1 |
| Kelso 1996 | CBA | USA<br>Teaching hospital and the community | N=39<br>women: 79.5%<br>mean age: 38.2<br>asthma: mod/sev | Comprehensive long-term management program for asthma in adult African-Americans<br>Ongoing<br>components: 6<br>main: EDU | Usual care | Process: 2<br>HC use: 2 |
| Saini 2004 | CBA | Australia<br>Pharmacies | N=67<br>women: 68.5%<br>mean age: 42.6<br>asthma: mod/sev | The Australia Six-Step Asthma Management Plan<br>6 months<br>components: 7<br>main: EDU | Usual care | Process: 1<br>HC use: 1<br>Pt satis: 1<br>QOL: 1<br>Symptoms/activity: 4<br>Self-care: 4 |
| Saini 2008 | CBA | Australia<br>Pharmacies | N=83<br>women: 69%<br>mean age: 50.6<br>asthma: mod/sev | Rural Asthma Management Service (RAMS)<br>6 months<br>components: 8<br>main: EDU | Usual care | Process: 2<br>HC use: 1<br>QOL: 1<br>Symptoms/activity: 2<br>Self-care: 4 |
| Tatis 2005 | CBA | USA<br>University-affiliated medical centre | N=396<br>women: 71%<br>mean age: 38.4<br>asthma: na | Culturally-targeted program aimed at reducing asthma morbidity<br>18 months<br>components: 8<br>main: EDU | Usual care | HC use: 2<br>QOL: 1<br>Self-care: 1 |
| Brandao 2009 | BA | Brazil<br>Outpatient clinics | N=102<br>women: 80.4%<br>mean age: 43<br>asthma: mod/sev | Program for the Control of Asthma and Allergic Rhinitis in Feira de Santana (ProAR-FS)<br>12 months<br>components: 4<br>main: EDU | Usual care before intervention | HC use: 2 |
| Charlton 1991 | BA | UK<br>PCPs | N=115<br>women: 62.3%<br>mean age: 56.2<br>asthma: mod/sev | Nurse-run asthma clinic<br>Ongoing<br>components: 8<br>main: EDU | Usual care before intervention | Process: 1<br>HC use: 1<br>Symptoms/activity: 3 |
| Charlton 1992 | BA | UK<br>PCPs | N=105<br>women: 57%<br>mean age: 42<br>asthma: na | Nurse-run asthma clinic<br>Ongoing<br>components: 4<br>main: EDU | Usual care before intervention | HC use: 3<br>QOL: 1<br>Pt satis: 1<br>Symptoms/activity: 3<br>Self-care: 1 |
| Dsouza 1994 | BA | New Zealand<br>Community centres | N=46 (at 12 mo)<br>women: 80%<br>mean age: 38<br>asthma: na | Wairarapa Asthma Program<br>4 months<br>components: 6<br>main: EDU | Usual care before intervention | Process: 3<br>HC use: 3<br>Symptoms/activity: 3<br>Lung function: 1 |
| Dsouza 1996 | BA | New Zealand<br>Hospital outpatient clinic | N=26 (at 12 mo)<br>women: 70%<br>mean age: na<br>asthma: mod/sev | Asthma Program<br>6 months<br>components: 6<br>main: EDU | Usual care before intervention | HC use: 1<br>Symptoms/activity: 4 |
| Emmerton 2003 | BA | New Zealand<br>Pharmacies | N=100<br>women: % na<br>mean age: na<br>asthma: na | Pharmaceutical care demonstration project<br>24 months<br>components: 6<br>main: EDU | Usual care before intervention | Process: 2<br>QOL: 2<br>Symptoms/activity: 3<br>Lung function: 1 |

| Study ID | Design (allocation) | Country HC setting | Patients | Intervention (name[1], duration, number of components, main component) | Comparison | Outcomes |
|---|---|---|---|---|---|---|
| Franco 2007 | BA | Brazil<br>Referral clinic | N=64<br>women: 84%<br>mean age: 45<br>asthma: mod/sev | Program for the Control of Asthma and Allergic Rhinitis (ProAR)<br>Ongoing<br>components: 6<br>main: ORG_HC | Usual care before intervention | HC use: 5<br>QOL: 1<br>Symptoms/activity: 1<br>Lung function: 2 |
| Franco 2009 | BA | Brazil<br>Referral clinic | N=180<br>women: 84%<br>mean age: 45<br>asthma: mod/sev | Program for the Control of Asthma and Allergic Rhinitis (ProAR)<br>Ongoing<br>components: 6<br>main: ORG_HC | Usual care before intervention | Process: 1<br>HC use: 4<br>QOL: 1<br>Symptoms/activity: 1<br>Lung function: 2 |
| Hertzman 2005 | BA | Russia<br>Hospitals and policlinics | N=85<br>women: 67%<br>mean age: 50.2<br>asthma: mod/sev | Ongoing<br>components: 7<br>main: EDU / ORG_HC | Usual care before intervention | HC use: 2<br>Pt satis: 1<br>Symptoms/activity: 4<br>Lung function: 1 |
| Ignacio-Garcia 2002 | BA | Spain<br>General hospital | N=63<br>women: 44%<br>mean age: 39.5<br>asthma: mod/sev | Asthma self-management education program<br>36 months<br>components: 7<br>main: EDU | Usual care before intervention | Process: 1<br>HC use: 3<br>Symptoms/activity: 5<br>Lung function: 1 |
| Licksai 2012 | BA | Canada<br>PCPs | N=312<br>women: 60.7%<br>mean age: 35.7<br>asthma: mild/mod | The Essex County Community Asthma Care Strategy<br>Ongoing<br>components: 7<br>main: ORG_HC | Usual care before intervention | Process: 4<br>HC use: 2<br>Symptoms/activity: 2 |
| Lind 2006 | BA | USA<br>Managed care organisation | N=1982<br>women: 66%<br>mean age: 35<br>asthma: mild/mod | The Washington State Asthma Program<br>Ongoing<br>components: 10<br>main: EDU | Usual care before intervention | Process: 8<br>HC use: 3<br>Pt satis: 1 |
| Lindberg 1999 | BA | Sweden<br>A PCP | N=63<br>women: 63.5%<br>mean age: na<br>asthma: mild/mod | Asthma Nurse Practice<br>12 months<br>components: 6<br>main: ORG_HC | Usual care before intervention | Process: 6<br>HC use: 1<br>Symptoms/activity: 2<br>Self-care: 1<br>Lung function: 3 |
| Maljanian 1999 | BA | USA<br>Hospital | N=68 (at 12 mo)<br>women: % na<br>mean age: na<br>asthma: mod/sev | Asthma Control and Education program (ACE)<br>12 months<br>components: 7<br>main: EDU | Usual care before intervention | HC use: 2<br>QOL: 2<br>Symptoms/activity: 1<br>Self-care: 1 |
| Mangiapane 2005 | BA | Germany<br>Pharmacies | N=128<br>women: 65.6%<br>mean age: 43.1<br>asthma: mild/mod | Community pharmacy-based pharmaceutical care<br>12 months<br>components: 5<br>main: EDU | Usual care before intervention | Process: 4<br>QOL: 1<br>Symptoms/activity: 3<br>Self-care: 4<br>Lung function: 4 |
| Nähri 2001 | BA | Finland<br>Pharmacies | N=26 (at 12 mo)<br>women: 75%<br>mean age: 41.3<br>asthma: mild/mod | Therapeutic Outcomes Monitoring (TOM) program<br>12 months<br>components: 5<br>main: EDU | Usual care before intervention | Pt satis: 2<br>QOL: 2<br>Self-care: 3 |
| Patel 2004 | BA | USA<br>Large medical group practice | N=2920 (at 12 mo)<br>women: % na<br>mean age: na<br>asthma: na | Advocate Health Centers (AHC) Asthma Project<br>12 months<br>components: 12<br>main:ORG_PT / ORG_HC | Usual care before intervention | Process: 4<br>HC use: 3<br>Self-care: 2 |
| Pauley 1995 | BA | USA<br>Hospital | N=25<br>women: 68%<br>mean age: 32<br>asthma: na | Asthma clinic<br>6 months<br>components: 5<br>main: EDU | Usual care before intervention | Process: 3<br>HC use: 2 |
| Ponte 2007 | BA | Brazil<br>Referral clinic | N=269<br>women: 75.8%<br>mean age: 46<br>asthma: mod/sev | Program for the Control of Asthma and Allergic Rhinitis (ProAR)<br>Ongoing<br>components: 5<br>main: ORG_HC | Usual care before intervention | HC use: 2<br>Symptoms/activity: 2 |

| Study ID | Design (allocation) | Country HC setting | Patients | Intervention (name[1], duration, number of components, main component) | Comparison | Outcomes |
|---|---|---|---|---|---|---|
| Steuten 2006 | BA | Netherlands<br>PCPs and a hospital | N=nc<br>women: 75.9%<br>mean age: 38.8<br>asthma: mild/mod | Regional disease management program<br>12 months<br>components: 7<br>main: ORG_HC | Usual care before intervention | HC use: 1<br>Pt satis: 1<br>QOL: 2<br>Lung function: 3 |
| To 2008 | BA | Canada<br>PCPs | N=551<br>women: 59%<br>mean age: 54<br>asthma: mild/mod | Primary care asthma pilot project (PCAPP)<br>12 months<br>components: 9<br>main: EDU | Usual care before intervention | Process: 4<br>HC use: 2<br>Symptoms/activity: 4 |
| Tschopp 2002 | BA | Switzerland<br>PCPs within a health network | N=66<br>women: 43.4%<br>mean age: 43<br>asthma: mod/sev | Mieux vivre avec son asthme<br>12 months<br>components: 9<br>main: EDU | Usual care before intervention | HC use: 3<br>QOL: 1<br>Symptoms/activity: 2<br>Lung function: 2 |
| Boonsawat 2013 | ITS (population based) | Thailand<br>Hospitals | N=44,124 (at baseline)<br>women: % na<br>mean age: na<br>asthma: na | National Asthma Program<br>Duration: nc<br>components: 7<br>main: ORG_HC | Usual care before intervention | Process: 2<br>HC use: 2 |
| Fireman 2004 | ITS (population based) | USA<br>Kaiser Permanente Medical group practices | 32,246 (patient years at baseline)<br>women: % na<br>mean age: 42<br>asthma: na | The Permanente Medical Group (TPMG) disease management program<br>12 months<br>components: 11<br>main: ORG_HC | Usual care before intervention | Process: 1<br>HC use: 5<br>Symptoms/activity: 2 |
| Haahtela 2006 | ITS (population based) | Finland<br>Hospitals, clinics and pharmacies | N=na<br>women: % na<br>mean age: na<br>asthma: na | Finnish National Asthma Program 1994-2004<br>Ongoing<br>components: 12<br>main: ORG_HC | Usual care before intervention | Org: 1<br>Process: 1<br>HC use: 5<br>Pt satis: 1<br>Symptoms/activity: 1 |
| Kauppi 2013 | ITS (population based) | Finland<br>Hospitals, clinics and pharmacies | N=na<br>women: % na<br>mean age: na<br>asthma: na | Finnish Allergy Program 2008-2018<br>Ongoing<br>components: ≥12<br>main: ORG_HC | Usual care before intervention | HC use: 1 |
| Mehring 2013 | ITS (population based) | Germany<br>PCPs | N=20,982 (at baseline)<br>women: % na<br>mean age: na<br>asthma: na | German disease management programs for asthma<br>Ongoing<br>components: 10<br>main: EDU / ORG_HC | Usual care before intervention | Process: 8<br>HC use: 1<br>Symptoms/activity: 1 |
| Souza-Machado 2010 | ITS (population based) | Brazil<br>Specialized clinics | N=na<br>women: % na<br>mean age: na<br>asthma: na | Program for the Control of Asthma and Allergic Rhinitis (ProAR)<br>Ongoing<br>components: 8<br>main: ORG_HC | Usual care before intervention | Process: 1<br>HC use: 1<br>Symptoms/activity: 1 |
| Carmo 2011 | XS | Brazil<br>Family health units | N=313<br>women: 57.2%<br>mean age: na<br>asthma: mild/mod | Respira Londrina<br>>4 months<br>components: 7<br>main: EDU | Limited intervention for patients | Org: 1<br>Process: 3<br>HC use: 3<br>Symptoms/activity: 1 |
| Lindberg 2002 | XS | Sweden<br>PCPs | N=348<br>women: 53.9%<br>mean age: 48.5<br>asthma: na | Asthma Nurse Practice<br>Duration: nc<br>components: 7<br>main: ORG_HC | Usual care | Process: 5<br>HC use: 1<br>QOL: 1<br>Symptoms/activity: 4<br>Self-care: 4 |

[1] name of intervention or brief description if name not provided RCT: randomised controlled trial; QRCT: quasi randomised controlled trial; CBA*: controlled before-after study (with 2 or more sites for intervention and control groups); CBA: controlled before-after study; ITS: interrupted-time-series study; BA: before-after study (without external control group); XS: cross-sectional studies (with concurrent measurement of intervention exposure and outcomes); PCP: primary care practice; EDU: education component; ORG_HC: organisational component targeting the healthcare system or professionals; ORG_PT: organisational component targeting patients; QoL: quality of life; HC: healthcare; na: not available; nc: not clear
